# Direct Observation of Franck–Condon Stimulated Emission and Sub-20 fs Relaxation in Photoexcited Flavins

**DOI:** 10.1021/acs.jpclett.6c00294

**Published:** 2026-03-28

**Authors:** Daniel Timmer, Krishan Kumar, Jan P. Götze, Peter Saalfrank, Antonietta De Sio, Christoph Lienau

**Affiliations:** ∇ Institut für Physik, Carl von Ossietzky Universität Oldenburg, 26129 Oldenburg, Germany; ‡ Institut für Chemie und Biochemie, Freie Universität Berlin, 14195 Berlin, Germany; § Institute of Chemistry, University of Potsdam, 14476 Potsdam, Germany; ∥ Center for Nanoscale Dynamics (CENAD), Carl von Ossietzky Universität Oldenburg, 26129 Oldenburg, Germany

## Abstract

Flavins are the chromophores in several blue-light-sensitive photoreceptor proteins and act as redox cofactors in many enzymes that are relevant for biological processes. Despite their biological relevance and numerous detailed optical investigations of their photophysical properties, the ultrafast nonequilibrium dynamics of their elementary optical excitations are not yet fully known. Here, we use ultrafast coherent vibrational spectroscopy with a 10-fs time resolution in the 450-nm spectral range to study their excited state coherent vibrational dynamics. We observe that coherent wavepacket motion along high-frequency C–C stretching modes with a ∼20-fs period is rapidly damped on a 20-fs time scale. In contrast, coherent motion along several low-frequency modes persists for much longer. We attribute this to a mode-selective intramolecular vibrational energy redistribution driven by nonadiabatic couplings between the optical bright state and a close-lying dark electronic state, in accordance with model calculations. Our results may be of relevance for the formation of long-lived radical pair states in magnetic-field sensitive proteins.

## Graphical abstract

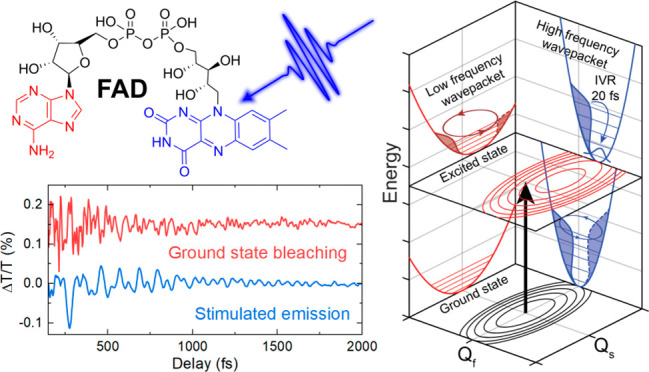

## Introduction

Flavins are prototypical blue-light-absorbing chromophores that act as cofactors for redox-active enzymes in various biological systems. Flavoproteins play a crucial biological function, such as DNA repair,^1,2^ regulation of circadian rhythm in plants^3^ and animals,^4,5^ as well as functions related to plant growth.^6,7^ Their isoalloxazine moiety, central to flavin-based cofactors such as riboflavin, flavin mononucleotide, and flavin adenine dinucleotide (FAD), is primarily responsible for their photophysical properties. FAD is a widespread cofactor in flavoproteins, such as BLUF (blue light sensing using FAD) proteins, flavin-containing monooxygenase, LOV (light, oxygen, voltage) proteins, and, in particular, blue-light-absorbing cryptochrome photoreceptors.^8–13^ Cryptochromes (Crys) fulfill a wide range of functions in plants and animals. Photoexcitation of cryptochrome triggers a series of electron transfer steps from nearby amino acid residues, forming long-lived, spin-correlated radical-pairs.^14–17^ Singlet–triplet interconversion of this radical pair is currently considered the main candidate for the microscopic origin of the magnetic field sense in migratory song birds.^15,18–20^ Indeed, such magnetic field sensitivity has been shown *in vitro* for European Robin cryptochrome 4 (*Er*Cry4).^21^ Flavin-based chromophores are also actively studied for photocatalytic transformation of small organic molecules, e.g., photooxidation of alcohols,^22,23^ cyclization of barbituric acid derivatives^24^ and amide bond formation between aliphatic or aromatic aldehydes and amines.^25^

Owing to their broad functional significance, the photophysical properties of flavins and flavoproteins have been extensively investigated.^16,26–34^ In particular, time-resolved experiments such as transient absorption (TA) and time-resolved fluorescence spectroscopy have been used to investigate the photophysical fate of the excited state. Time-resolved fluorescence spectroscopy has shown strongly quenched emission from the stacked conformation between adenine and the isoalloxazine ring in FAD.^31,35–37^ The strong emission quenching has been largely^32^ assigned to an intramolecular electron transfer,^31,38–40^ and the influence of pH on the excited state has been investigated using TA.^41,42^ TA has also been used to explore the dynamics of the different redox states of FAD^21,28^ that exist in the planar or bent isoalloxazine ring structure, with the latter resulting in faster excited state deactivation.^16,31^ Furthermore, stimulated Raman spectroscopy has been used to investigate the vibrational modes of FAD in the excited state.^33^ This study also points to a central role of vibronic couplings in intramolecular vibrational energy redistribution (IVR) in the optically excited states.

Flavins show a broad absorption spectrum in the blue (Figure 1b) with two apparent electronic transitions, a lower-energy band close to 450 nm and an additional band in the UV close to 370 nm. Theoretical studies of the electronic structure^43–48^ assign these bands to the two lowest-energy ππ* transitions in the molecule. While the 450-nm band is commonly associated with the *S*_0_→*S*_1_ transition, different labeling of the higher-lying electronic states is found in the literature. We follow ref 47 and associate the 370-nm band with the *S*_0_→*S*_4_ transition (see Table 1 in ref 47). Electronic structure calculations give evidence of two additional, optically dark *n*π* transitions to states *S*_2_ and *S*_3_ in this energy region. The energy spacing between *S*_1_ and the closest, higher-lying *S*_2_ state is strongly solvent dependent.^33,49^ Mixed quantum classical calculations predict that nonadiabatic couplings between *S*_1_ and *S*_2_ play a fundamental role in the excited state dynamics in flavins. They suggest that such couplings are driven by high-frequency (∼22-fs) excited state vibrations.^47^ They induce a strong red-shift of the stimulated emission spectra within the first few 10 fs after excitation and result in rapid IVR processes, dampening high-frequency vibrational wavepacket motion in <100 fs.^47^ Recently, related nonadiabatic dynamics have been discussed theoretically in cryptochromes,^50,51^ and low-lying conical intersections were predicted in time-dependent density functional theory (TD-DFT) simulations of lumiflavin.^52^ So far, direct time-domain studies of the role of such nonadiabatic couplings in the excited state dynamics of flavin systems are lacking. Quite generally, experimental studies of nonadiabatic dynamics in solution and in thin films with a time resolution faster than the period of the relevant vibrational modes are limited.^53–58^

**Figure 1:**
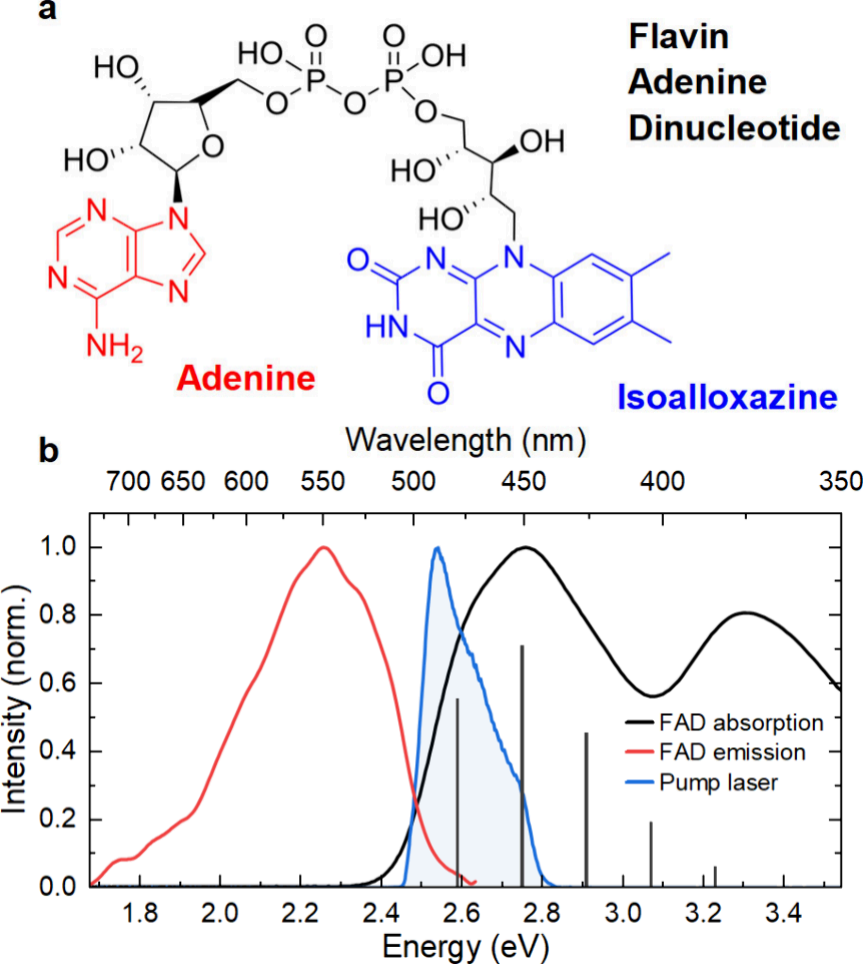
(a) Chemical structure of flavin adenine dinucleotide (FAD), consisting of a blue-light-absorbing isoalloxazine moiety that is connected to adenine via a ribityl chain. (b) Normalized steady-state absorption spectrum of FAD in water (black), showing the dominant *S*_0_ → *S*_1_ transition at ∼2.8 eV and the *S*_0_ → *S*_4_ transition at ∼3.3 eV. A faint vibronic progression with a dominant 160 meV vibrational mode is observed in the lower-energy band, as indicated by a stick spectrum. The red-shifted emission spectrum is shown in red. The spectrum of the pump pulses (blue) is chosen to overlap with the two vibronic resonances of FAD in the S_1_ electronic state.

Here, we report on the experimental observation of nonadiabatic couplings in FAD by directly following the excited state dynamics using TA spectroscopy with high time-resolution. Utilizing 12-fs blue excitation pulses, we observe that the vertically excited Franck–Condon state decays within ∼20 fs accompanied by the ultrafast formation of a red-shifted stimulated emission band. Our results suggest that nonadiabatic couplings result in the rapid loss of coherent high-frequency vibrational motion in the excited state, while oscillations resulting from low-frequency modes persist on the picosecond time scale. Our results reveal how vibronic coupling shapes excited state dynamics in a key biological cofactor, with implications for understanding the light-driven function in flavoproteins.

## Results and Discussion

As in all flavins, the optically relevant moiety in FAD is an isoalloxazine motif with its center ring connected to an adenine dinucleotide unit through a ribityl chain (Figure 1a). The steady state absorption spectrum of an aqueous solution of FAD (Figure 1b, black line) shows the typical flavin absorption in the blue spectral region close to 2.75 eV (450 nm) that can be assigned to the *S*_0_→*S*_1_ electronic transition of the isoalloxazine moiety, primarily involving excitation from a π_2_ highest occupied molecular orbital (HOMO) to the π_3_ lowest unoccupied molecular orbital (LUMO).^48^ A second absorption band close to 3.35 eV (370 nm) has been assigned to a transition from S_0_ → S_4_ involving excitation from HOMO–1 to LUMO.^48,59–62^ Theoretical calculations show that, in addition to these two optically bright excited states, two dark states *S*_2_ and *S*_3_ with *n*π* character lie energetically close to *S*_1_.^43–46^ The *S*_0_ → *S*_1_ absorption band in Figure 1b exhibits three faint shoulders close to 2.59 eV (∼480 nm), 2.75 eV (∼450 nm), and 2.91 eV (∼426 nm). We assign these shoulders to |*S*_0_,0⟩ → |*S*_1_,*n*⟩ vibronic transitions, where *n* = 0, 1, 2 denotes the quantum number of a high-frequency, ∼160-meV (∼1290 cm^–1^) vibrational mode (Figure 1b, stick spectrum). This progression, more pronounced in a protein environment than in aqueous solution,^14^ is in excellent agreement with a previously computed absorption spectrum.^59^ The experimental emission spectrum of FAD in water (Figure 1b, red line) shows an essentially mirrored, red-shifted emission band centered around 2.25 eV (∼550 nm).

### Ultrafast TA Spectroscopy of FAD

To experimentally investigate the role of coherent vibrational dynamics and, in particular, the effects of nonadiabatic couplings on these dynamics, we perform transient absorption (TA) spectroscopy of a 200 μM aqueous solution of FAD. To generate ultrashort and broadband excitation pulses, we developed a 10-kHz TA setup based on hollow-core fiber supercontinuum pulses. While the broadband pulses^63^ are directly used as the probe, a 4f spectral filter is employed to prepare tunable pump pulses, centered around 470 nm (Figure 1b, blue line). These pulses are compressed using chirped mirrors to a pulse duration of 12 fs (see Methods). The pulse compression substantially improves on the time resolution reached in earlier studies of flavins,^2,16,30–32,40,49,64–66^ including our own work based on an optical parametric amplifier.^14,67^ The aim is to resolve coherent oscillations in the electronic ground and excited states of all expected Raman-active modes of FAD, including C–C stretching modes in the ∼1500 cm^–1^ range. The pump pulse mainly overlaps with the ground and the first excited vibronic state in the *S*_0_ → *S*_1_ transition of FAD (see Figure 1b). Due to the ultrashort pulse duration and low molar extinction coefficient of FAD (11300 M^–1^ cm^–1^),^68,69^ the TA data exhibit significant cross-phase modulation (XPM) during pulse overlap.^70^ To minimize XPM contributions and uncover dynamics for short delays,^71,72^ the broadband probe is also compressed with chirped mirrors, and a reference measurement of the bare solvent is recorded under identical experimental conditions. The obtained Δ*T*/*T* map of FAD is presented in Figure 2a after careful chirp correction and solvent subtraction (Supporting Information, section 3). The TA map shows a ground state bleaching (GSB) band centered at around 2.75 eV, a stimulated emission (SE) band centered at around 2.25 eV, and three excited state absorption (ESA) bands centered at around 2.45 eV, above 3 eV, and below 2 eV (Figure 2a). This assignment of the bands is well established in the literature.^33,73^

**Figure 2:**
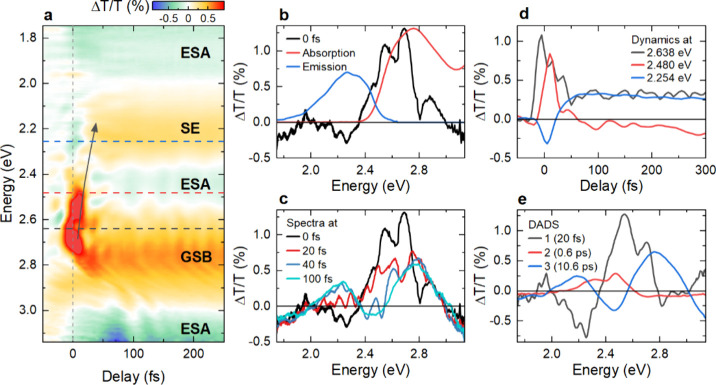
Transient absorption spectroscopy of FAD dissolved in water. (a) Map of differential transmission Δ*T*/*T* spectra of FAD as a function of the pump–probe delay and probe energy. The 12-fs pump pulses are centered around 2.6 eV (Figure 1). Important regions in the map are associated with ground-state bleaching (GSB), stimulated emission (SE), and excited-state absorption (ESA) transitions. SE from the Franck–Condon region, initially centered around 2.6 eV, displays a rapid red-shift during the first 50 fs (black arrow). This red-shift is taken as the signature of rapid intramolecular vibrational energy redistribution (IVR) in FAD. (b) Comparison of Δ*T*/*T* spectrum at *t* = 0 fs (black) with the scaled steady-state absorption (red) and emission (blue) spectrum from Figure 1. (c) Δ*T*/*T* spectra at selected delays, highlighting the transient red-shift of the SE and the rapid buildup of a relaxed SE band centered around 2.2 eV. (d) Δ*T*/*T* dynamics at selected probe energies reveal the rapid decay of SE from the Franck–Condon region (black), the transient red-shift (red), and the buildup of the relaxed SE (blue). (e) Decay-associated differential spectra (DADS) obtained by a global analysis. The corresponding decay times are given in the legend.

The Δ*T*/*T* spectrum at t = 0 fs, recorded during impulsive optical excitation, is depicted as a black line in Figure 2b. The spectrum is centered at around 2.6 eV, and its line shape is somewhat similar to the steady state absorption spectrum of FAD in water (red line). The TA spectrum at t = 0 fs shows two dominant peaks at 2.55 and 2.69 eV, slightly red-shifted by ∼70 meV compared to energies of the |*S*_1_,0⟩ ← |*S*_0_,0⟩ and |*S*_1_,1⟩ ← |*S*_0_,0⟩ transitions in the linear absorption spectra. It is difficult to reconcile the 2.55 eV peak with the |*S*_1_,0⟩ → |*S*_0_,1⟩ transition, which should be red-shifted in energy by one vibrational quantum with respect to the |*S*_1_,0⟩ → |*S*_0_,0⟩ transition. We tentatively assign the 70 meV red-shift to the quasi-instantaneous electronic solvation of flavin upon optical excitation in the aqueous solvent.^74^ Thus, the two dominant peaks we observe in the TA spectrum are likely due to the |*S*_1_,0⟩ → |*S*_0_,0⟩ and |*S*_1_,1⟩ → |*S*_0_,0⟩ SE transitions. This implies that the SE stems from an essentially unrelaxed Franck–Condon region in the excited state. This is supported by observing negligible spectral intensity at lower energies in the region of the emission spectrum (blue line). This TA spectrum undergoes a rapid temporal evolution during the first 100 fs after excitation (Figure 2c). The strong initial SE around 2.6 eV vanishes rapidly within 50 fs, and a red-shifted SE around 2.2 eV builds up. This SE band, well reported in the literature,^14,32,33,40,49^ displays no further significant spectral evolution after 100 fs, and its amplitude decays slowly on a time scale of several ps. Around 2.75 eV, the long-lived GSB band remains after the initial 100-fs relaxation.

To further analyze the early time evolution, Δ*T*/*T* transients are plotted at selected probe energies in Figure 2d. The Δ*T*/*T* signal in the low-energy part of the GSB region, at 2.64 eV (black line), shows a fast decay with <50 fs. The signal at 2.48 eV (red line) rapidly builds up within 10 fs and then decays within <50 fs, leaving behind a long-lived ESA signal. In the SE region, around 2.25 eV (blue line), the TA signal shows a delayed rise within <50 fs (blue line).

We globally fit^14,67,75^ the Δ*T*/*T* spectra to quantify these observations. We obtain three exponential decays in the experimental time window of 3 ps (Supporting Information, section 3). The corresponding decay-associated difference spectra (DADS) are presented in Figure 2e. As expected, the long-lived, 10.6-ps DADS component shows contributions from a GSB, a SE and an ESA band, matching earlier reports.^14,32,33,40,49^ The associated decay component of 10.6 ps extends beyond the measured time window and, therefore, reflects an effective long-time decay of the signal. Earlier studies report multiexponential decay dynamics with a component representing the ns lifetime of open conformer FAD and a ∼5-ps intramolecular electron transfer in stacked conformer FAD.^14,31,39,40^ The 0.6-ps component mainly reflects small spectral shifts and has been assigned to solvation processes.^14,40^ Interestingly, the first, so far unreported, DADS has two spectral components. A positive part is centered around 2.6 eV, which resembles the absorption spectrum of FAD (Figure 2b) and reflects SE from the vertically excited Franck–Condon region. The negative part around 2.2 eV is associated with the buildup of the red-shifted SE band. The analysis suggests a short lifetime of this first component of only 20 fs. We assign this decay time to the rapid evolution of the stimulated emission spectrum immediately after photoexcitation.

### Vertical Excitations from DFT/MRCI

To confirm our assertion that the TA spectrum immediately after photoexcitation (black line in Figure 2b) shows ESA and SE from the unrelaxed Franck–Condon region, we performed DFT and subsequent DFT/MRCI (multireference configuration interaction DFT, see Methods section) simulations of riboflavin in water. Riboflavin was chosen over the full FAD to reduce computational cost, since the lowest excited states are located at the flavin part of FAD. The obtained spectra are shown in Figure 3 and in Tables S2 and S3 in the Supporting Information. The linear absorption (Figure 3, black line) shows two strong low-energy transitions at 2.75 and 3.37 eV as well as two weak transitions at 3.06 and 3.40 eV, in line with earlier reports,^47,48,59,76,77^ which assign the two intense peaks (here *S*_1_ and *S*_3_) to (ππ*) transitions from *S*_0_, and the two others in this energy range (here *S*_2_ and *S*_4_) to (*n*π*) transitions. Note, however, a change of state order between the energetically very close S_3_ and S_4_, compared to earlier reports obtained with TD-DFT,^47^ which indicate *S*_4_ to be a bright state, contrasting the DFT/MRCI results. However, the states from the previous work are not 1:1 comparable to the present results; DFT/MRCI includes multielectron excitation character, which affects in particular higher states, here leading to the “swapping” of *S*_3_, *S*_4_ states. Including solvation is important, as it is known from earlier work^47^ that the lowest bright states are red-shifted by up to 0.2 eV when introducing water. The SE from the Franck–Condon point peaks at 2.75 eV (Figure 3a, red line), coinciding with the *S*_0_ → *S*_1_ transition in absorption. The SE from the relaxed *S*_1_ geometry is significantly red-shifted by ∼400 meV (Figure 3b, red line), as a consequence of rapid intramolecular structural reorganization on the *S*_1_ potential energy surface. We also calculated the ESA spectrum (Figure 3a, blue line) at the Franck–Condon point, showing mainly three bands, in line with our experimental TA spectra (Figure 2a). In contrast to the rapid evolution of SE, these ESA bands are much less affected by intramolecular structural reorganization. This supports our assumption that the early time TA spectrum (black line in Figure 2b) indeed contains the contributions of ESA and SE from the unrelaxed Franck–Condon region.

**Figure 3:**
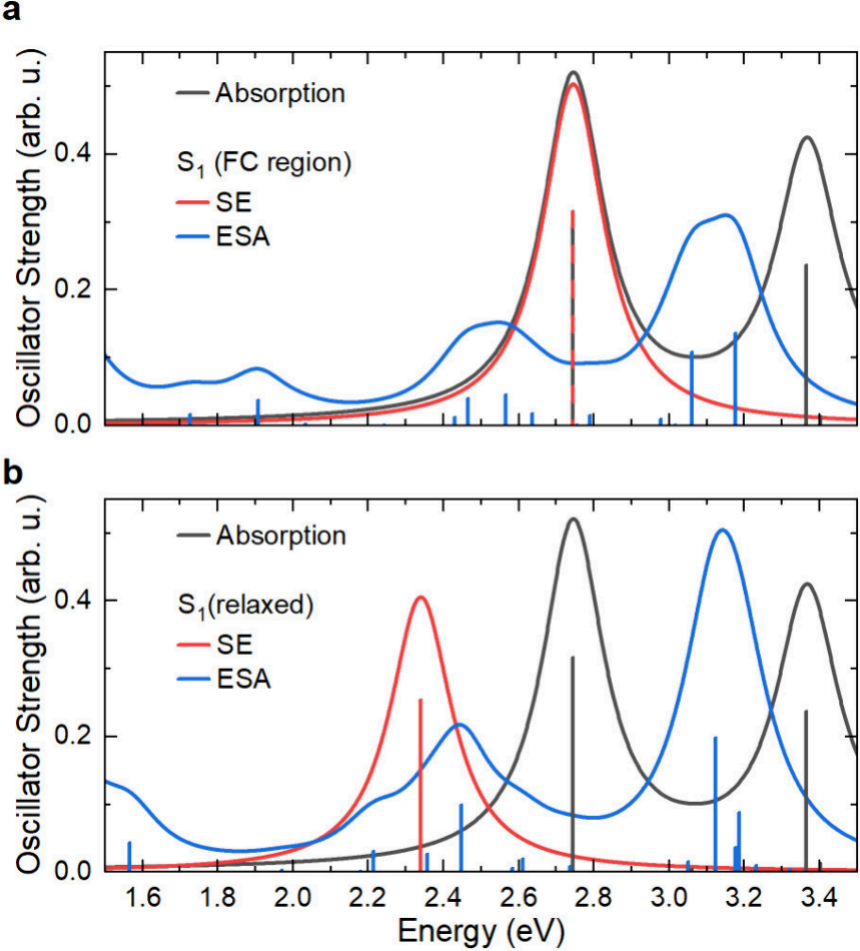
DFT/MRCI calculations; optical spectra of riboflavin. (a) Linear absorption (black), SE (red, identical to the *S*_1_ linear absorption peak), and ESA spectrum (blue) simulated in the unrelaxed molecular geometry at the Franck–Condon (FC) point. (b) Spectra calculated from a relaxed geometry in the S_1_ state (SE, red; ESA, blue; linear absorption, black, as in (a) for comparison). Note the substantial red-shift of the SE band (red line). Lorentzian line shapes with a line width of 200 meV (full width at half-maximum) are assumed for all transitions, as well as a scaling factor of 0.9 for all energies to improve agreement to experimental data.

### Analysis of Coherent Vibrational Dynamics

It is evident from Figures 2a,d, that the transient dynamics also show pronounced oscillations, reflecting the coherent vibrational motion of the molecule. In particular, high-frequency (∼25 fs) modulations can be seen in the GSB region (Figure 2d, black line), while they are essentially absent in the SE band (Figure 2d, blue line). Slower oscillations can also be seen, especially in the region between SE and GSB. To understand the influence of coherent vibrational wavepacket motion on the excited state dynamics, we quantitatively analyze the spectral dependence of these oscillations.

To isolate these coherent modulations, we subtract the exponential decay dynamics from the raw data (Supporting Information, section 3). In Figure 4a, the resulting residuals are shown as an example at selected probe energies in the GSB (2.695 eV) and SE regions (2.254 eV). In the SE region, the residuals reveal only slow oscillations with periods of 50 to 120 fs. In the GSB region, instead, high-frequency oscillations with periods down to 20 fs are also visible. To reveal the vibrational frequencies of the slow and fast modes, we perform a Fourier transform (FT) analysis of these residuals along the delay axis (Figure 4b). Below 1000 cm^–1^, we observe the same dominant low-frequency modes in the SE and GSB regions, while high-frequency modes in the 1100–1700 cm^–1^ range are present in the GSB region only. To correlate the oscillation amplitudes of these modes with the different contributions to the TA signal (Figure 4c), we show the GSB, SE and ESA components obtained from the DFT/MRCI calculations in Figure 3c. We also plot a map of the FT spectra as a function of the probe energy in Figure 4d. A FT spectrum obtained by integrating the data in Figure 4d along the probe energy axis with ∼10 cm^–1^ resolution is shown in Figure 4e. The resulting mode frequencies are in quantitative agreement with reported Raman spectra, as compared in Table S1 of the Supporting Information.^78–80^

**Figure 4:**
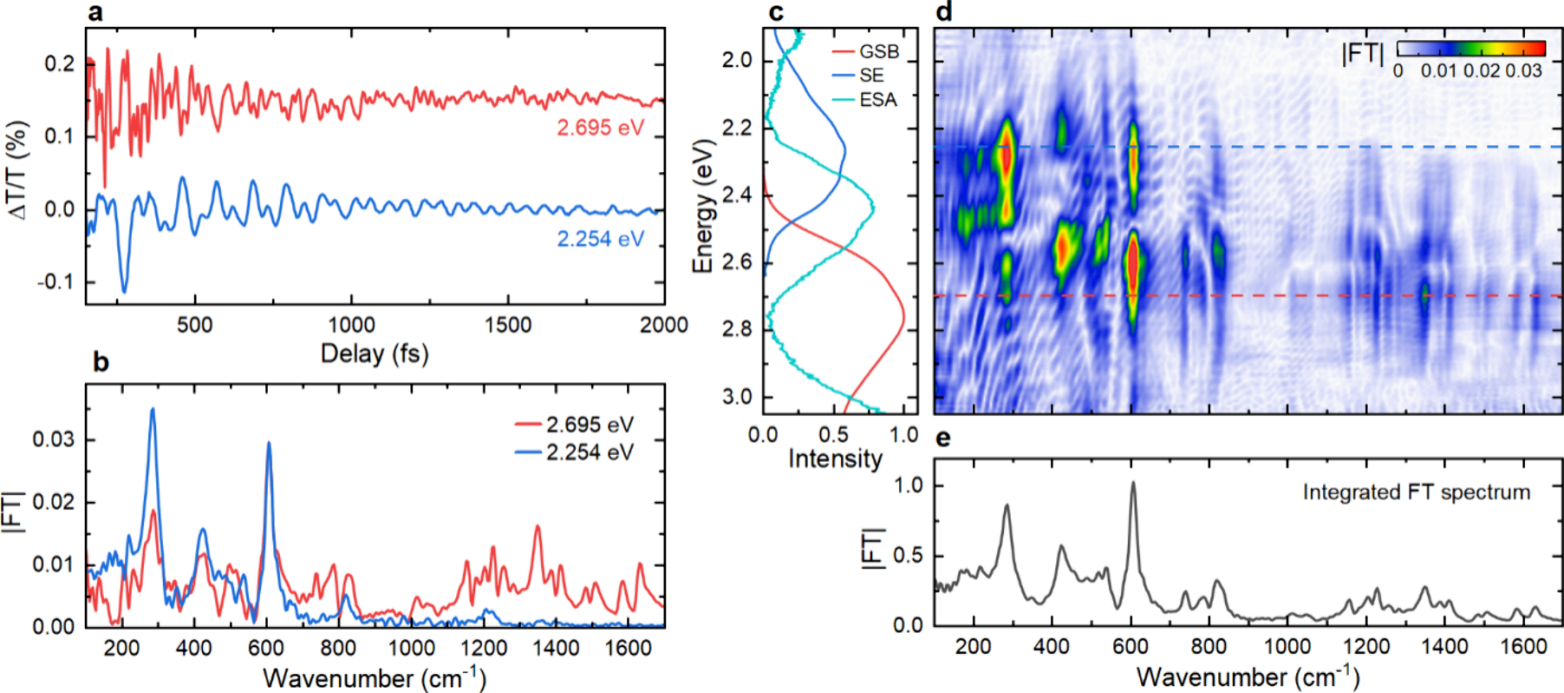
Fourier transform (FT) analysis of coherent vibrational dynamics in FAD dissolved in water. (a) Residuals from the GSB region (2.695 eV) and SE region (2.254 eV) were obtained by subtracting time-dependent DADS from the experimental Δ*T*/*T* data. The residuals have been vertically shifted for clarity. (b) Comparison of the FT spectra deduced from the residuals in (a). High-frequency components (>1000 cm^–1^) are absent in the SE spectra (blue), while the dominant low-frequency modes show similar amplitudes. (c) Decomposition of the quasi-static DADS (blue line in Figure 2e, not shown here) into GSB (red), SE (blue), and ESA (cyan) components, assuming that the line shapes of the GSB and SE components match those observed in linear spectroscopy (Figure 1). (d) Two-dimensional map of the FT amplitude as a function of the mode frequency and probe energy. The dashed lines mark the FT spectra plotted in (b). (e) FT spectrum obtained by integrating the map along the probe energy. The integrated spectrum contains all coherently excited ground and excited state modes and is in good agreement with the reported Raman spectra of FAD in water.

Importantly, the FT map clearly reveals that the high-frequency modes are primarily localized in the GSB region, centered around the 0–0 transition close to 2.6 eV. Their amplitude vanishes in the SE region. The probe energy dependence of all high-frequency modes is very similar, as shown in Figures S9 and S10 in the Supporting Information. In stark contrast, the low-frequency modes display more diverse spectral profiles. Generally, these profiles, discussed in more detail in Figure 5, are shifted toward lower probe energies and overlap with the SE and ESA regions. Additionally, some of the low-frequency modes, in particular, 285 cm^–1^, 424 cm^–1^, and 607 cm^–1^, show substantially larger amplitudes than all high-frequency modes.

**Figure 5:**
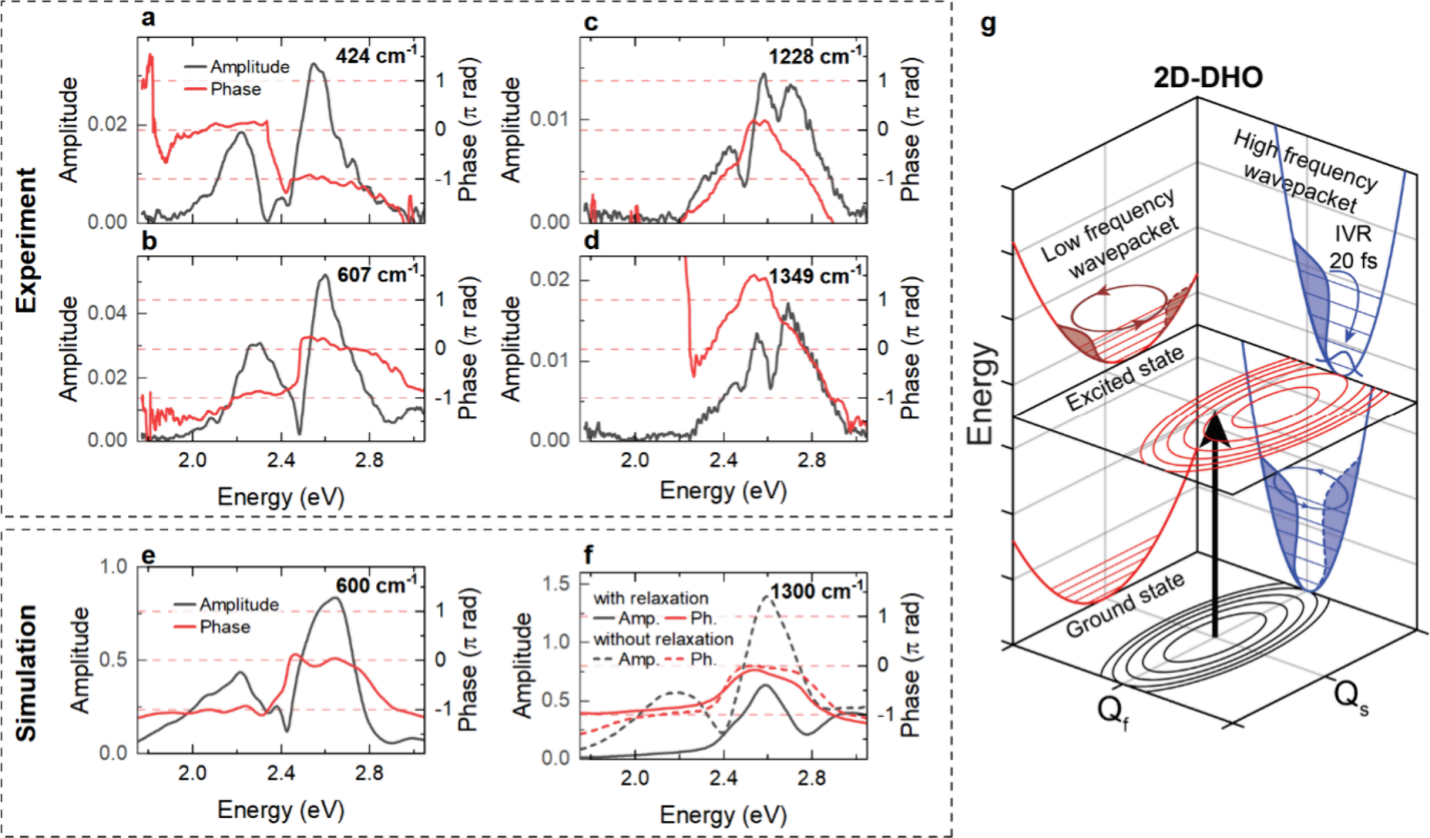
Probe energy dependence of the FT analysis in Figure 4 for selected high- and low-frequency modes of FAD. (a-d) Amplitude (black) and spectral phase profiles (red) for the 424 cm^–1^ (a), 607 cm^–1^ (b), 1228 cm^–1^ (c), and 1349 cm^–1^ (d) modes obtained by FT analysis of the coherent Δ*T*/*T* oscillations. (e-f) Amplitude (black) and phase profiles (red curve) for a 600 cm^–1^ (e) and 1300 cm^–1^ (f) mode simulated using a two-dimensional displaced harmonic oscillator (2D-DHO) model. For the high-frequency mode (f), simulations in the absence (dashed) and presence (solid) of rapid 20-fs excited state vibrational relaxation are reported, whereas relaxation is neglected for the low-frequency mode. With relaxation, the amplitude vanishes in the SE region, in good agreement with the experimental results in panels (b) and (d). (g) Sketch of the relaxation scenario in a 2D-DHO model. Impulsive optical excitation launches a vibrational wavepacket in the excited-state Franck–Condon region (black arrow). IVR leads to rapid relaxation along the high-frequency *Q*_*f*_ coordinate, while coherent oscillations persist along *Q*_*s*_. In contrast, ground state wavepacket oscillations are launched along *Q*_*f*_, but they are much weaker along *Q*_*s*_.

We want to argue that the high-frequency oscillations are only seen in the GSB region, because they correspond to wavepacket motion in the *S*_0_ electronic ground state. In contrast, the low-frequency oscillations also appear in the SE/ESA region because they are triggered in the excited *S*_1_ state. For this, we report the probe energy dependence of the amplitude and phase of the complex Fourier spectra of several selected modes in Figure 5. These data are shown for two selected low-frequency modes, 424 cm^–1^ (b) and 607 cm^–1^ (c), and two high-frequency modes at 1228 cm^–1^ (d) and 1349 cm^–1^ (e). The amplitude profiles of both high-frequency modes are centered in the GSB region and exhibit two distinct minima at 2.48 and 2.63 eV, each coinciding with a π phase jump. No significant amplitude is observed in the SE region between 2.0 and 2.3 eV. In contrast, the low-frequency modes exhibit substantial amplitude in both the SE region and the GSB region. The phase profiles of the low-frequency modes show differences. The 607 cm^–1^ mode displays a phase jump from −π to 0 around the amplitude dip at 2.48 eV. In contrast, the 424 cm^–1^ mode undergoes a phase jump from 0 to −π around 2.4 eV.

To rationalize these observations, it is instructive to recall detailed experimental^33,49^ and theoretical studies^47^ of the ultrafast coherent dynamics of flavin molecules. Weigel et al.^49^ have used TA spectroscopy with 30-fs pulses to study such dynamics. They observed the SE band already during their time resolution and have assigned its formation to an ultrafast (<20-fs) ππ*–*n*π* vibronic coupling process. In later work, Weigel et al.^33^ applied Femtosecond Stimulated Raman Spectroscopy to access the vibrational modes in the ground *S*_0_ and excited *S*_1_ states of riboflavin and FAD in different solvents. The reported Raman bands have been analyzed and assigned using quantum-mechanical normal-mode analysis. Generally, the observed mode frequencies are in very good agreement with our observations (Supporting Information, Table S1). Also in this work, time-resolved fluorescence and TA spectra recorded for delays of >100 fs reveal the presence of a SE around 2.25 eV during the experimental time resolution. In a theoretical study by Klaumünzer et al.^47^ the nonadiabatic dynamics of riboflavin in the gas phase and in a microsolvated water environment were calculated using a mixed quantum classical surface hopping approach. Their calculations show the appearance of a distinct, high-energy SE band reflecting emission from the Franck–Condon region shortly after photoexcitation. The line shape of this band is similar to the linear absorption spectrum and shows vibronic substructure resulting from the coupling to C–C or C–N stretching modes with a 22-fs period. This high-energy SE is present for no more than 10 fs. The excited state wavepacket motion that is associated with this mode decays rapidly within <50 fs, resulting in the buildup of a strongly red-shifted SE band. This red-shift is more pronounced in the microsolvated environment. These ultrafast dynamics reflect a very rapid IVR process in riboflavin resulting from vibronic coupling of the bright ππ* and the dark *n*π*states that is induced by these high-frequency vibrational modes.

In accordance with our simulations shown in Figure 3, this suggests that the strong Δ*T*/*T* band around 2.65 eV in Figure 2b indeed reflects the SE from the Franck–Condon region immediately after photoexcitation. The pronounced red-shift (Figure 2a, black arrow) is now readily understood. It likely reflects an exceptionally fast IVR process in FAD due to the strong nonadiabatic coupling in flavins. This IVR process seems to be complete within 50 fs since we do not observe significant changes in the red-shifted SE spectrum after this redistribution process. IVR also explains the absence of high-frequency vibrational oscillations in the red-shifted SE region in Figure 4b,d since it quickly dampens high-frequency vibrational motion in the excited state. The low-frequency modes, instead, appear to be unaffected by this IVR process.

### Displaced Harmonic Oscillator Simulations

To test how such rapid IVR manifests in our TA spectra, we set up the conceptually simplest phenomenological model that can account for the coherent oscillations in our spectra. We choose a model of two harmonic oscillators that are coupled to an electronic two-level system, as sketched in Figure 5g. We simulate TA signals based on this 2-mode DHO model by nonperturbatively solving the Lindblad master equation for the density matrix of our system^72^ to account for vibrational energy relaxation in the *S*_1_ state and for pure electronic dephasing. We simulate the effects of ground and excited state vibrational dynamics launched by a 12-fs pulse centered around 2.64 eV. To account for the linear absorption spectrum of FAD, we consider a single, high-frequency mode with an effective energy of *ℏ*ω_*vib*,1_ = 160 meV (∼1300 cm^–1^) and dimensionless displacement of Δ_1_ = 1.6. For the second mode, we choose *ℏω*_*vib*,2_ = 74 meV (∼600 cm^–1^) and Δ_2_ = 1.2 (Supporting Information, section 6). With this, we can indeed qualitatively reproduce the experimental absorption (Figure S12) and emission spectra. Obviously, our simplified DHO model does not capture the full Stokes shift that is seen in the experiment since it neglects the structural reorganization in the *S*_1_ state that is demonstrated by the calculations in Figure 3. When simulating the TA dynamics, we introduce a finite vibrational relaxation time of 20 fs for the high-frequency mode in the electronically excited state. This entirely phenomenological relaxation time is chosen to qualitatively account for the rapid damping of the high-frequency vibrational wavepacket and cannot capture the complex, multimode nonadiabatic relaxation process.

In such a DHO model, coherent vibrational wavepackets are launched in the excited state after impulsive excitations via a vertical Franck–Condon transition.^81,82^ The wavepacket motion can be read out via SE transitions showing periodic modulations with the frequencies of the two modes and wavepacket oscillation amplitudes that increase with mode displacement.^83^ Ground state vibrational wavepackets are created via impulsive stimulated Raman scattering^84^ and read out via GSB transitions.^83^ For short excitation pulses, the wavepacket oscillation amplitude decreases linearly with pulse duration since a propagation of the excited state wavepacket is needed to reach a finite displacement in the ground state.^82^ For our 12-fs pump pulses, this implies that wavepackets along both vibrational coordinates are launched into the excited state (Figure 5g). In contrast, ground state wavepacket motion is predominantly excited along the high-frequency coordinate while the displacement of the low-frequency wavepacket is much weaker. TA maps resulting from these 2D-DHO simulations are shown in Figures S13 and S14. They show a SE spectrum with a shape that mirrors the absorption and displays a Stokes shift of ∼400 meV (Figure S12). This Stokes shift is significantly smaller than the experimental one observed in Figure 1b since intramolecular structural relaxation (Figure 3) is neglected.

The simulated TA maps show persistent oscillations at the frequencies of both vibrational modes. An analysis of the spectral mode profiles of the Fourier transforms of these oscillations is particularly instructive. The mode profile for the low-frequency mode (Figure 5e) is essentially independent of the relaxation time of the excited state high-frequency mode. In agreement with experiment (Figure 5b), it shows a large amplitude in both the GSB (2.6 eV) and SE (2.3 eV) regions. The amplitude dip at ∼2.4 eV coincides with a phase jump from −π to 0. As in experiment, the amplitude abruptly drops for energies above 2.8 eV, i.e., where the SE is strongly reduced. This is the signature that the low-frequency wavepacket is predominantly launched in the excited state and is read out via SE. While this DHO model can reproduce the amplitude and phase profiles for the 607 cm^–1^ mode, the 424 cm^–1^ mode shows qualitatively different behavior, in particular the different sign of the phase jump. This suggests that here, the excited state wavepacket is not read out via SE but rather via an ESA to a higher-lying electronic state. Similar phase signatures are also seen for several other low-frequency modes (see Figures S9 and S10).

In the absence of vibrational relaxation, the spectral mode profile of the 1300 cm^–1^ mode is rather similar to that of the 600 cm^–1^ mode, except for a significantly larger amplitude at energies above 2.8 eV, i.e., on the high-energy side of the GSB band (Figure 5f, dashed line). The reason for this difference becomes evident when vibrational relaxation is turned on (Figure 5f, solid line). Now, the spectral amplitude in the SE region below 2.3 eV vanishes, and the amplitude between 2.4 and 2.8 eV is reduced by half. Now, the SE contribution of the excited state wavepacket to the spectral profile is absent. Above 2.8 eV, however, the SE amplitude is negligible, and the probe pulse can sense only the ground state wavepacket that is launched by stimulated impulse Raman scattering. Therefore, the amplitude in this high-energy region remains unchanged when altering the relaxation rate for the high-frequency mode, since only ground state wavepacket motion is probed.

Despite the simplicity of the 2D-DHO model, the simulated spectral mode profiles in the presence of rapid vibrational relaxation are in reasonable agreement with experiment. In contrast, those calculated without relaxation show a high amplitude in the 2.3-eV SE region, which is not observed in the experimental data.

## Summary and Conclusion

In summary, we have observed, immediately after impulsive photoexcitation of FAD molecules in aqueous solution, a short-lived stimulated emission band from the Franck–Condon region of the electronically excited *S*_1_ state. This band decays within 50 fs, resulting in the buildup of a substantially red-shifted, relaxed stimulated emission band. Our data show that this rapid relaxation process is concurrent with an essentially complete damping of coherent oscillations of all high-frequency (>1000 cm^–1^) modes in the electronically excited state, while low-frequency, excited-state wavepacket motion persists for up to 1 ps. Our results align well with quantum-dynamical simulations for riboflavins, which support the assignment of our TA spectra and relate this mode-selective and fast intramolecular vibrational energy redistribution process to nonadiabatic couplings between the optically bright *S*_1_ state and an energetically close-lying dark state,^47^ mediated by the high-frequency carbon backbone modes of the isoalloxazine moiety.

As such, our results may be of direct relevance for closely related FAD-binding cryptochrome proteins for which nonadiabatic couplings between *S*_1_ and *S*_2_ have recently been predicted theoretically.^50^ This raises the question which role such nonadiabatic couplings play for the dynamics and yield of charge transfer processes^14^ and radical pair formation^21^ in these magnetically sensitive proteins. Experiments in this direction are currently underway in our laboratory. More generally, our results present an example of very rapid IVR processes driven by nonadiabatic couplings that are caused by (a multitude of) high-frequency vibrational modes. It appears to us that the quantum dynamics of such processes and their functional relevance for charge-separation processes in molecules and nanosystems are currently understood only to a limited extent, at least experimentally.^53^ Pump–probe and, in particular, two-dimensional electronic spectroscopy with few-fs or even sub-fs time resolution may provide desirable new insight.

## Methods

### Ultrafast Pump–Probe Spectroscopy

Ultrafast pump–probe experiments are conducted using a home-built setup based on hollow-core fiber supercontinua (see Figure S1).^63^ A 1-m hollow-core fiber (Savanna, Ultrafast Innovations) filled with 2.4 bar Neon gas (absolute pressure) is pumped by a regenerative Ti:sapphire laser (Legend Elite, Coherent) that outputs 26-fs pulses with 1 mJ pulse energy centered at 800 nm with 10 kHz repetition rate. The generated supercontinuum spectrum, spanning ∼350–1000 nm,^63^ is filtered to a range of ∼390–720 nm and split into a pump and probe beam. While the probe is used directly, the pump is further spectrally filtered in the spectral domain using a 4f setup. Pump spectra are set to cover a range from ∼435–500 nm using a tunable slit in the Fourier plane of the 4f setup. Chirped mirrors (DCM12, Laser Quantum) are used to compensate for dispersion of the pump and probe. This yields a pump pulse duration of 12 fs as measured via transient grating frequency-resolved optical gating (see Figure S2). The delay between pump and probe *t*_*d*_ is set via a retroreflector mounted on a motorized translation stage (M112.1DG, Physik Instrumente), and both beams are focused into the 1 mm quartz sample cuvette using an off-axis parabolic mirror to ∼40 μm spot size. The transmitted probe beam is spectrally dispersed, and spectra *S*_*pu*,*pr*_(λ) are recorded with full laser repetition rate using a fast and sensitive line camera (Aviiva EM4, e2v). Mechanical chopping of pump and probe using a custom chopper blade with 2:1 duty cycle allows recording of scattering-corrected differential transmission spectra

1 $$\frac{\Delta T}{T}\left(t_{d},\lambda \right)=\frac{S_{\mathrm{on},\mathrm{on}}-S_{\mathrm{off},\mathrm{on}}-S_{\mathrm{on},\mathrm{off}}}{S_{\mathrm{off},\mathrm{on}}}$$

from all combinations of blocked (off) and transmitted (on) pump and probe.^63^ Polarizations of the pump and probe are set via thin-film polarizers.

For all experiments, flavin adenine dinucleotide (FAD) as purchased from Sigma-Aldrich is dissolved in water with a concentration of 200 μM and experiments are performed at room temperature. For each data set, a solvent reference measurement of bare water is performed under identical experimental conditions. The experimental data recorded with compressed probe pulses are shown in Figure 2. To avoid any spurious effects of higher-order phases resulting from probe compression, we additionally record TA data with an uncompressed, chirped probe (shown in Figures 4 and 5) for quantitatively analyzing spectral mode profiles.

For the first data set recorded with a compressed probe, the relative polarization between pump and probe is set to the magic angle (54.7°), and a pump pulse energy of 25 nJ is used. For the second data set with chirped probe, the relative polarization is tuned parallel and a pump pulse energy of 20 nJ is used. Pump–probe data are recorded with 5-fs step size up to a delay of 3 ps. More details can be found in the Supporting Information sections 1 and 2.

### Data Evaluation

For both data sets, pump–probe transients of FAD and the water reference contain a strong cross-phase modulation^70^ (XPM) signal centered around zero delay. This XPM is used to determine the wavelength-dependent time zero arising from the chirp of the probe pulse, and all measurements are shifted accordingly. Using the solvent reference, the XPM contribution is carefully subtracted from the FAD data (Supporting Information, section 3). A global analysis is performed on the data using a Matlab-based toolbox,^14,67,75^ yielding decay-associated difference spectra. For analyzing the coherent vibrational dynamics, the fit obtained from the global analysis is subtracted from the data to obtain residuals. A Fourier transform (FT) of these residuals yields complex FT maps as a function of the vibrational frequency and probe energy. Additionally, spectral mode profiles in amplitude and phase can be obtained for each vibrational mode by taking crosscuts through the FT map. For more details, see Supporting Information section 3.

### DFT Calculations

The riboflavin model was built starting from the protein data bank entry for FAD, conveniently labeled “FAD”, and then removing the structure to riboflavin by replacing the first phosphorus atom with hydrogen and deleting all subsequent atoms in the structure. The structure was then relaxed using the CAM-B3LYP^85^ functional and the def2-TZVP^86^ basis set using the ORCA^87^ software, employing an implicit water solvation (CPCM).^88^ The most recent version of the DFT multireference configuration interaction (DFT/MRCI)^89^ approach, the R2022 Hamiltonian, was then used to obtain the spectra depicted in Figure 3, based on a BHLYP^90^ orbital guess with the def2-SVP basis. Energies were found to be slightly overestimated; thus, we employed a scaling of 0.9 to improve agreement with experimental values.

For relaxed riboflavin (TD-DFT^91^ optimization of the *S*_1_ state, in CPCM), the same DFT/MRCI procedure (see previous paragraph) was applied; the optimization started from the Franck–Condon point.

## Supplementary Material



## Data Availability

The experimental data supporting the claims in this study are presented in the manuscript and in the Supporting Information in graphic form and can be obtained from the authors upon reasonable request.

## Notes

Experimental details, data evaluation, vibrational mode profiles, comparison with literature, simulation details, additional simulation results, Figures S1–S14, Tables S1–S4. (PDF)
