# Supplementary material for: Direct Observation of Franck–Condon Stimulated Emission and Sub-20 fs Relaxation in Photoexcited Flavins
Source: J Phys Chem Lett. 2026 Mar 28;17(14):4114–24. doi: 10.1021/acs.jpclett.6c00294 (PMC13071916; doi:10.1021/acs.jpclett.6c00294)
Supplement: Supplementary file 1 [file jz6c00294_si_001.pdf]

Supporting Information for

Direct Observation of Franck–Condon Stimulated Emission and Sub-20 fs Relaxation in Photoexcited Flavins

Daniel Timmer[a+], Krishan Kumar[a+], Jan P. Götze[b], Peter Saalfrank[c], Antonietta De Sio[a,d], and Christoph Lienau[a,d,*]

[a] Institut für Physik, Carl von Ossietzky Universität Oldenburg, 26129 Oldenburg, Germany

[b] Institut für Chemie und Biochemie, Freie Universität Berlin, 14195 Berlin, Germany

[c] Institute of Chemistry, University of Potsdam, 14476 Potsdam, Germany

[d] Center for Nanoscale Dynamics (CENAD), Carl von Ossietzky Universität Oldenburg, 26129 Oldenburg, Germany

*Correspondence to: christoph.lienau@uni-oldenburg.de

+Both authors contributed equally.

Content

1. Experimental setup 2
2. Experimental conditions 3
3. Data evaluation 4
   Time zero correction 4
   Removal of XPM contribution 4
   Global analysis 6
   Fourier analysis 7
4. Vibrational modes 7
   Comparison with literature 7
   Additional spectral mode profiles 9
5. DFT/MRCI calculations 11
6. Simulation of pump-probe signals 12
   Numerical modelling 13
   Lindblad operators 14
   Simulation parameters 14
   Simulation results 15
7. References 18

1. Experimental setup

Commonly employed pump-probe setups based on narrowband excitation using optical parametric amplifiers[1, 2] and broadband chirped supercontinuum probe pulses as generated in, e.g., CaF_2 are usually limited in their time resolution in the blue wavelength range to ~30-50 fs. Such pulses do not allow to resolve coherent vibrational dynamics of high-frequency modes with periods in the 20-fs range. We therefore set up a pump-probe experiment based on a hollow-core fiber supercontinuum[3] to improve the temporal resolution.

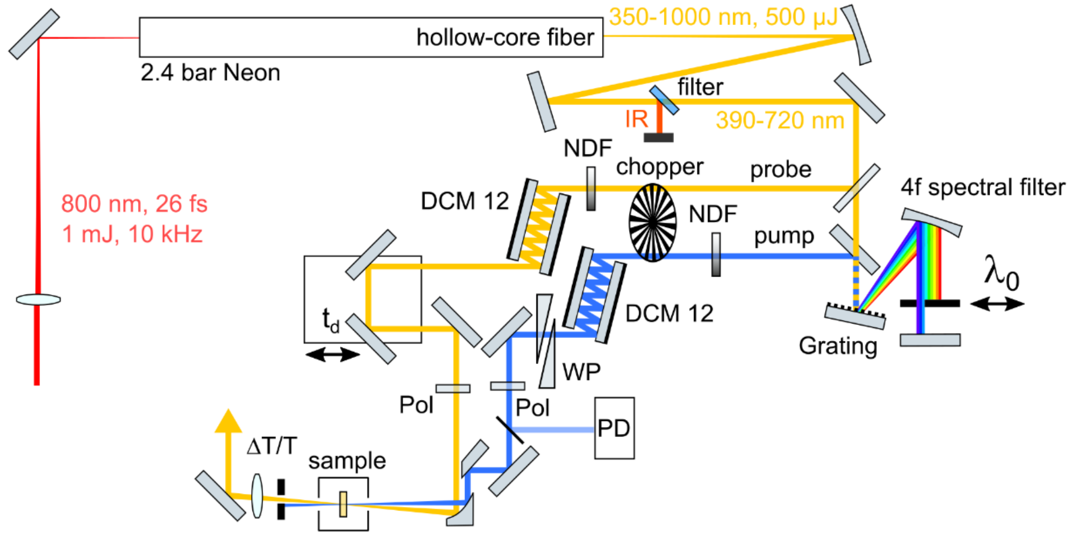

Figure S1: Experimental pump-probe setup based on a hollow-core fiber supercontinuum and 4f spectral filtering. NDF = neutral density filter, PD = photo diode, WP = wedge pair, Pol = polarizer.

The setup, schematically shown in Fig. S1, uses a regenerative amplifier (Legend Elite, Coherent) seeded by a titanium-sapphire oscillator (Vitara, Coherent). The amplifier system outputs 1-mJ pulses centered at 800 nm at 10 kHz repetition rate with a pulse duration of ~26 fs. A white light supercontinuum spanning from ~350-1000 nm is generated in a 1-m hollow-core fiber (Savanna, Ultrafast Innovations) using an absolute pressure of 2.4 bar neon gas.[3] The hollow-core fiber is optimized to achieve broad spectral coverage and good spatial beam profile while maintaining acceptable single-shot spectral stability (Fig. S2b).[3] After removing the infrared contribution using a dichroic filter (700 nm Low GDD Dichroic Shortpass Ultrafast Filter, Edmund Optics) a small fraction of the ~390-720 nm spectrum is split off to be used as the probe pulse and is compressed using chirped mirrors (DCM12, Laser Quantum). The remaining part is used as the pump and is spectrally filtered using a 4-f setup based on a reflection grating (GR25-0305, 300 lines/mm blazed at 500 nm, Thorlabs) and cylindrical mirror (f = 100 mm) and a movable slit is used to select the desired spectral components in the Fourier plane. This allows for convenient and easy adaptation of both the spectral bandwidth and the center wavelength. Pump and probe are both chopped (MC2000B, Thorlabs) with a custom-made chopper blade with a 2:1 duty cycle to allow for shot-to-shot data acquisition that includes scattering correction.[3] Pump pulses are compressed using chirped mirrors (DCM12, Laser Quantum) and a movable wedge pair. Thin-film polarizers (WGF HC11N, Edmund optics) are used to set the polarization of pump and probe and a variable neutral density filter to set the excitation power. A small fraction of the pump pulse is fed to a 10 MHz photo diode that is synchronized with the laser system to read out the current chopper state and monitor pump power. The delay between the pump and probe t_d is tuned with a retro reflector mounted on a motorized translation stage (M112.1DG1, Physik

Instrumente). A 150 mm focal length off-axis parabolic mirror focuses both pulses into a 1 mm quartz cuvette to ~40-µm spot size and the transmitted probe beam is recollimated and sent to a grating spectrograph (Acton SP-2150, 150 lines/mm grating blazed for 300 nm, Princeton Instruments). A fast and sensitive line camera (Aviiva EM4, e2v) is used to record spectra $S(\lambda)$ as a function of wavelength at full 10-kHz laser repetition rate. From a series of three consecutive probe spectra the scattering-corrected differential transmission

$$\frac{\Delta T}{T}(t_d, \lambda) = \frac{S_{\text{on,on}} - S_{\text{off,on}} - S_{\text{on,off}}}{S_{\text{off,on}}} \tag{S1}$$

is computed, where $S_{pu,pr}$ denotes the spectrum recorded for a chopper state of the pump (pu) and probe (pr) which can be either transmitted (on) or blocked (off).

2. Experimental conditions

Flavin adenine dinucleotide (Sigma Aldrich) is dissolved in water with a concentration of 200 µM as confirmed by UV-VIS absorption measurements using a molar extinction coefficient of 11300 M^{-1} cm^{-1} of FAD.[4] Water without any additions is used as reference for the pump-probe studies.

To achieve effective excitation of the flavin (see Fig. 1) the spectrum is tuned to the blue spectral range to cover ~435-500 nm, achieving high time resolution while reducing excess power in a spectral region that does not excite the sample. Contributions below ~435 nm are avoided since the employed chirped mirrors (DCM12, Laser Quantum) do not sufficiently compress these spectral components.[3] A home-built transient grating frequency resolved optical gating[5] (TG-FROG) setup is used to characterize the excitation pulses. A measured TG-FROG is shown in Fig. S2a, yielding a retrieved pulse duration of ~12 fs.

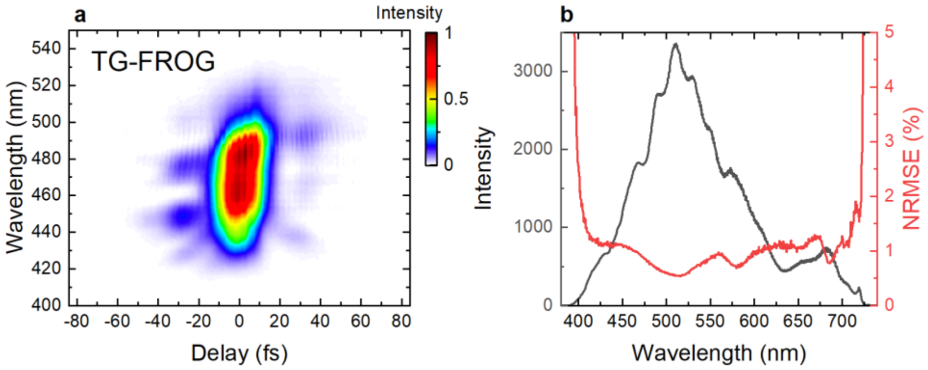

Figure S2: *Pulse duration and laser stability. a) TG-FROG trace yielding a pulse duration of ~12 fs. b) Spectral single shot stability measured for 1000 consecutive laser pulses using the Aviiva line camera showing a normalized root mean square error of ~1%.*

For all experiments, a step size of 5 fs is used and 1000 laser spectra are averaged per time step for delays up to 3 ps. Multiple such pump-probe scans are recorded, drifts in time zero are corrected, and averaged.

We record two datasets with slightly different experimental approaches to be able to analyze different aspects of the FAD dynamics. For the dataset shown in Fig. 2, the setup depicted in Fig. S1 is used where chirped mirrors (DCM12) are compressing the probe pulses in order to reduce effects of cross-phase modulation[6] (XPM) on the early <50 fs dynamics. Here, a pump pulse energy of 25 nJ is used and the polarization between pump and probe is set to the magic angle.

To allow for a more quantitative analysis of the vibrational mode profiles, minimizing possible effects of higher-order sinusoidal phases of the probe pulse, data presented in Figs. 3 and 4 are recorded with a

slightly modified setup where the probe pulse is not compressed and remains chirped. As a result, a more pronounced and temporally extended XPM signal hinders access to the early <50 fs dynamics. A pump pulse energy of 20 nJ is used with pump and probe polarization set to parallel.

3. Data evaluation

Time zero correction

For all measurements, a corresponding solvent (water) reference is recorded under identical experimental conditions using the same parameters. This allows to correct (to first order) for strong XPM contributions during pump-probe pulse overlap not originating from FAD. Since the XPM signal is around an order of magnitude stronger than the flavin nonlinearity, it can be used both in the sample and reference measurement to determine the wavelength-dependent time zero. Uncorrected raw data for FAD and the reference measurements are shown in Fig. S3 and Fig. S4 together with the deduced time zero (black lines).

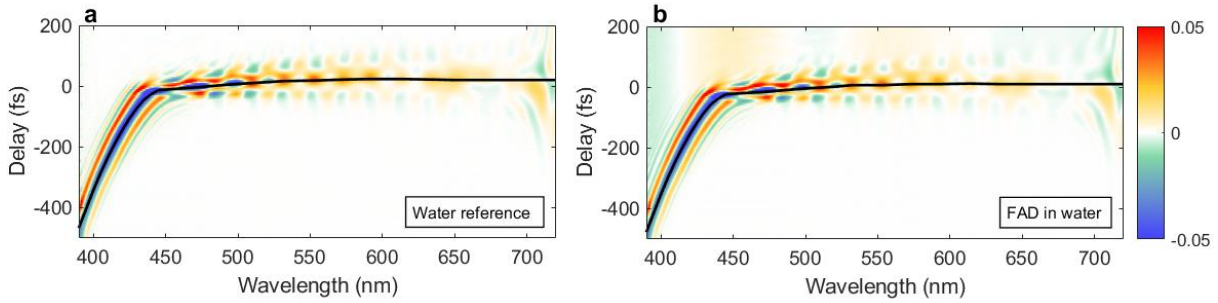

Figure S3: *Correction of time zero for data using a compressed probe and magic angle polarization. a) Uncorrected pump-probe map of the water reference measurement. b) Uncorrected pump-probe map of FAD in water. In both cases, the time zero, deduced from the XPM signal, is marked as black lines. For wavelength below ~435 nm, the employed chirped mirrors do not compensate the spectral phase and a remaining chirp of the probe pulse can be seen.*

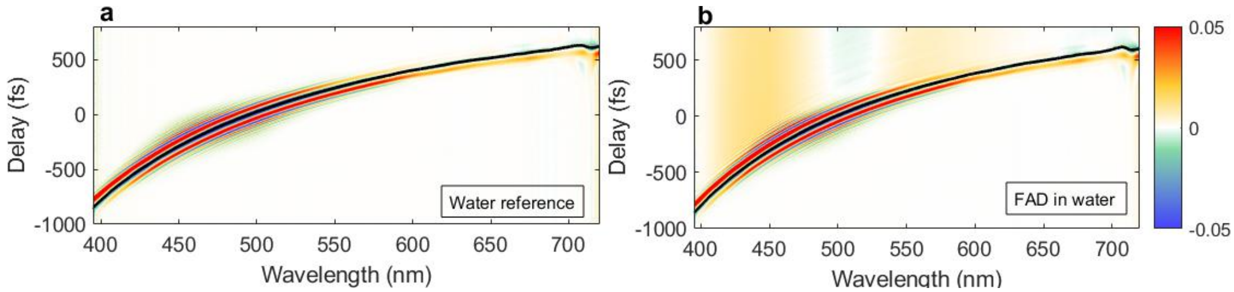

Figure S4: *Correction of time zero for data using a chirped probe and parallel polarization. a) Uncorrected pump-probe map of the water reference measurement. b) Uncorrected pump-probe map of FAD in water. In both cases, the time zero, deduced from the XPM signal, is marked as black lines.*

Removal of XPM contribution

Using the deduced time zero, the FAD data are shifted accordingly and resampled onto the original time axis with 5 fs step size. The shift of the reference measurement is further refined with sub-fs precision for each wavelength to optimize XPM removal before resampling. The shifted reference measurement is then subtracted from the FAD data with a wavelength-dependent amplitude $A(\lambda)$ (between 0.9 and 1) that is again determined to minimize residual XPM contributions.

Fig. S5 shows the time zero corrected reference data (black lines) and that for FAD before (red lines) and after (blue lines) subtraction for selected wavelengths for the dataset using the compressed probe. In both

cases, the XPM signal is centered around $t_d = 0$ and allows for accurate determination of time zero within approximately one step size of 5 fs.

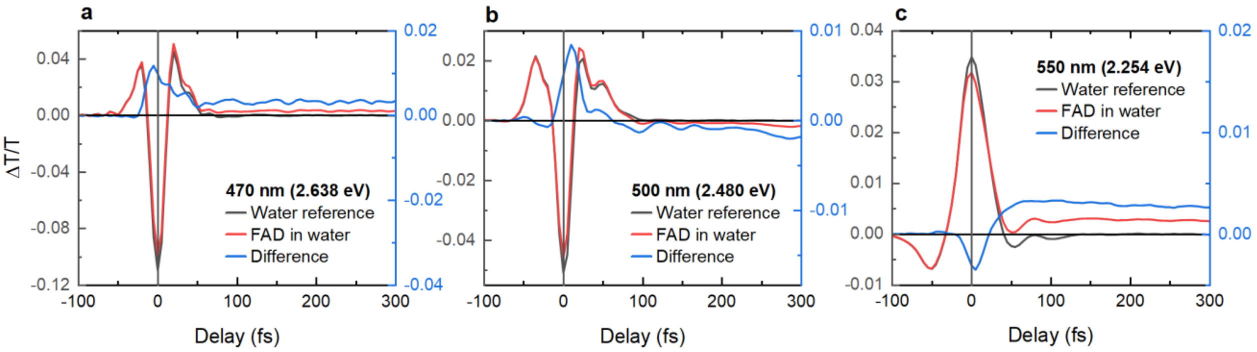

Figure S5: Removal of XPM contribution for selected wavelengths of a) 470 nm, b) 500 nm and c) 550 nm. A water reference measurement (black lines) is used to record the XPM contribution under identical experimental conditions. Before correction, the same strong XPM signal centered around $t_d = 0$ is also contained in the FAD data (red lines). After subtraction (blue lines), the ultrafast FAD dynamics are isolated. For clarity, the XPM-corrected data (blue lines) is enhanced (right y-axis).

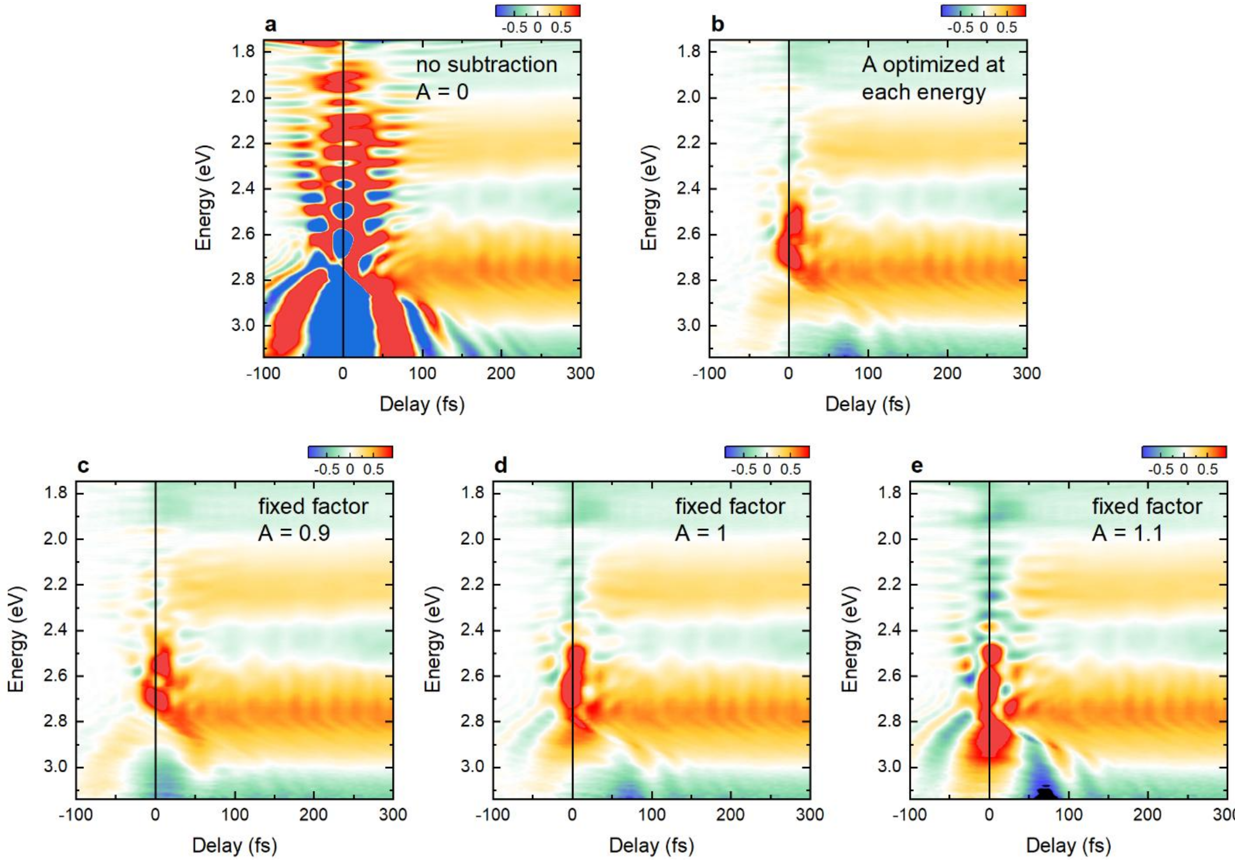

Figure S6: Effect of XPM subtraction factor on FAD pump-probe map. a) Time zero corrected pump-probe map for FAD. Around zero delay, strong XPM is visible. b) XPM-corrected map as shown in Fig. 2a using an optimized subtraction factor $A(\lambda)$. c-e) Maps using a fixed factor of 0.9 (c), 1.0 (d) and 1.1 (e). Already for a factor of 0.9 and 1.1 residual XPM contributions can be discerned as spectral modulations around zero delay.

To ensure that the observed dynamics during the early <50 fs are not a spurious result of the XPM correction procedure, Fig. S6 shows the effect of the subtraction amplitude A on the pump-probe maps,

now plotted as function of the probe energy. Without correction (Fig. S6a), strong XPM is seen centered around zero delay. The subtraction with optimized amplitude $A(\lambda)$ is shown in Fig. S6b and is the same data as already displayed in Fig. 2a. The two main observations are the rapid decay of signal at zero delay in the region between 2.4-2.8 eV and rise of the stimulated emission band in the 2.0-2.4 eV range within ~20 fs. Fig. S6c-e show that these observations are robust against choosing also fixed subtraction amplitudes of 0.9, 1.0 and 1.1, respectively. While the map obtained for a fixed amplitude $A = 1$ closely resembles that seen in Fig. S6b, already a slight increase or decrease of the subtraction amplitude by 10% results in clearly visible XPM contributions seen as oscillatory spectral modulations around zero delay, in particular in the region close to the stimulated emission signal. Reduction of these XPM structures is used to optimize $A(\lambda)$. Considering the comparatively strong XPM signal (Fig. S6a), the near absence of any such XPM structure in Fig. S6b compared with those seen for constant A in Fig. S6c,e demonstrates proper XPM correction and therefore allows for analyzing the remaining signal as arising predominantly from FAD. Due to the strong XPM amplitude, faint residual spectral oscillations superimposed onto the FAD signal during the first ~100 fs cannot be avoided.

Data recorded with the chirped probe are corrected analogously. However, the nature of the XPM and slight mismatches between the FAD and reference measurement in this case do not allow for such clean solvent correction as shown above.

Global analysis

After the time zero and solvent correction, the data are evaluated using a global analysis using a Matlab-based toolbox.[2, 7] Data starting at $t_d = 0$ are used for the dataset obtained with compressed probe, while the first 100 fs are discarded in case of the chirped probe as they are still contaminated by residual XPM contributions.

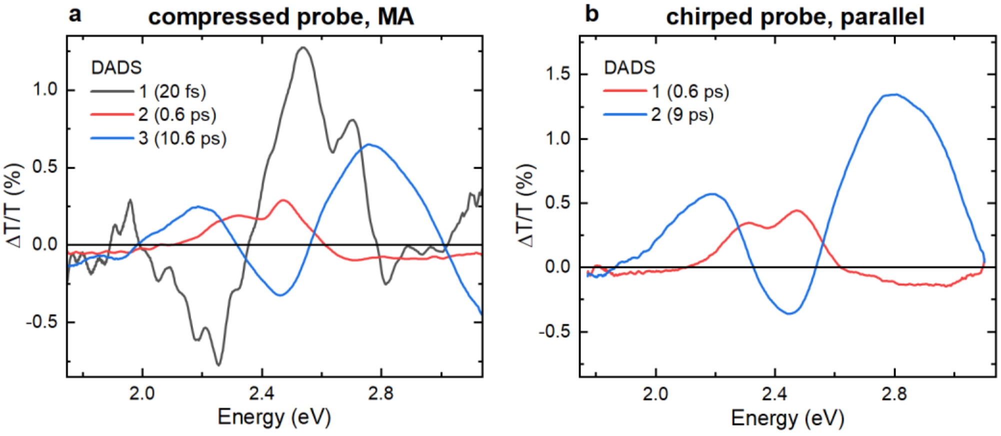

Figure S7: *DADS obtained from global analysis. a) Compressed probe dataset using magic angle polarization. b) Chirped probe dataset using parallel polarization.*

For the compressed probe dataset, three components are needed to fit the data. The resulting decay-associated difference spectra (DADS) are shown in Figs. S7 and 2 and the associated decay times are 20 fs, 0.6 ps and 10.6 ps. The second and third DADS reproduce reasonably well those reported for FAD.[2, 8] The 0.6 ps component (DADS2) has been associated with solvation dynamics,[8] while the 10.6 ps component (DADS3) captures multiple slower dynamics. Due to the limited 3 ps measurement range, slower multi-exponential dynamics are represented by an effective third component. We expect for FAD three additional components of around 5 ps, 30 ps and 3 ns, reflecting intramolecular electron transfer from

adenine to isoalloxazine in the stacked FAD conformer, dimer dynamics and open conformer decay, respectively.[2, 8] The first 20 fs component (DADS1) has not been reported so far in the literature and reflects the rapid intramolecular vibrational energy redistribution (IVR) leading to a red-shift of the stimulated emission.

For the data obtained with the chirped probe, only two components are required for the global analysis since the IVR dynamics are not contained in the cut delay range. The DADS and associated decay times of 0.6 ps and 9 ps, shown in Fig. S7b, agree well with DADS2 and DADS3 of the compressed probe dataset and those expected from literature. [2, 8]

Fourier analysis

For analyzing the coherent vibrational dynamics, we compute residuals by subtracting the results of the global analysis from the experimental XPM-corrected data. Subsequent Fourier transform (FT) along the delay axis results in FT maps showing the vibrational spectrum for each detection energy. For this, the first 200 fs are discarded (data set to zero) due to remaining contamination from XPM contributions that lead to artifacts in the Fourier spectra. The data are zero-padded to 8 ps and a Gaussian filter with 4 ps full-width at half-maximum is applied. This analysis allows for quantitative analysis of the spectral mode profiles for each vibrational mode in amplitude and phase.

4. Vibrational modes

Detection energy integrated spectra of the FT amplitude in Fig. S8 show that both datasets using the chirped probe (black line) and compressed probe (red line) give very comparable results.

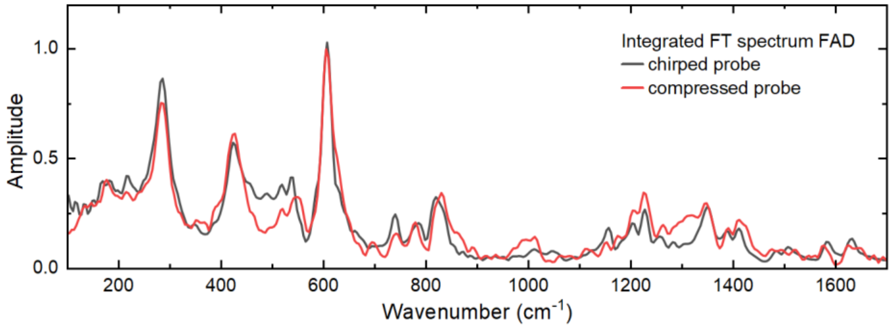

Figure S8: *Spectrally integrated Fourier spectra for the two datasets using the chirped probe (black line) and compressed probe (red line). Both approaches result in very comparable FT spectra.*

Comparison with literature

The frequencies of the modes observed in the experiment with the chirped probe are listed in table S1 and compared to published Raman modes of FAD.[9-12] Good agreement, typically better than 10 cm^{-1}, between coherent modes seen in our pump-probe data and published Raman spectra can be observed.

Table S1: *Vibrational modes extracted from the dataset using chirped probe compared to literature spectra for FAD_{ox}. Modes below 1066 cm^{-1} are readouts from FAD/H_20 data plotted in figure 8 of Ref* **[12]**.

Our data	Merk et al. (Ref [11])	Schelvis et al. (Fig. 3 in Ref [10])	Copeland et al. (Ref [9])	Weigel et al. (Ref [12])
1632	1625	1626	1634	1632
1585	1579	1580	1584	1584
-	1550	1554	1552	1548
1509	1505	1500	1505	1503
1484	1480	1458	1483	1462
1412	1422	1405	1426	1409
1388	1373	-	-	-
1349	1332	1355	1340	1354
1308	1305	1299	1310	
1283				1283
1258	1252	1247	1259	1256
1228	1223	1231	1232	1230
1201	-	-	-	
	-	1181	1183	1182
1154	1160	1160	1169	1160
1047	-	-	1071	1066
1012	1008	-		
834	-	830		831
818	-	-		
782	790	789		788
739	732	743		741
	685	678		
607	609	629		610
		600		
538		535		530
518		?		
489				
424		431		428
348		346		
		315		
285				296
				217
185				184

Additional spectral mode profiles

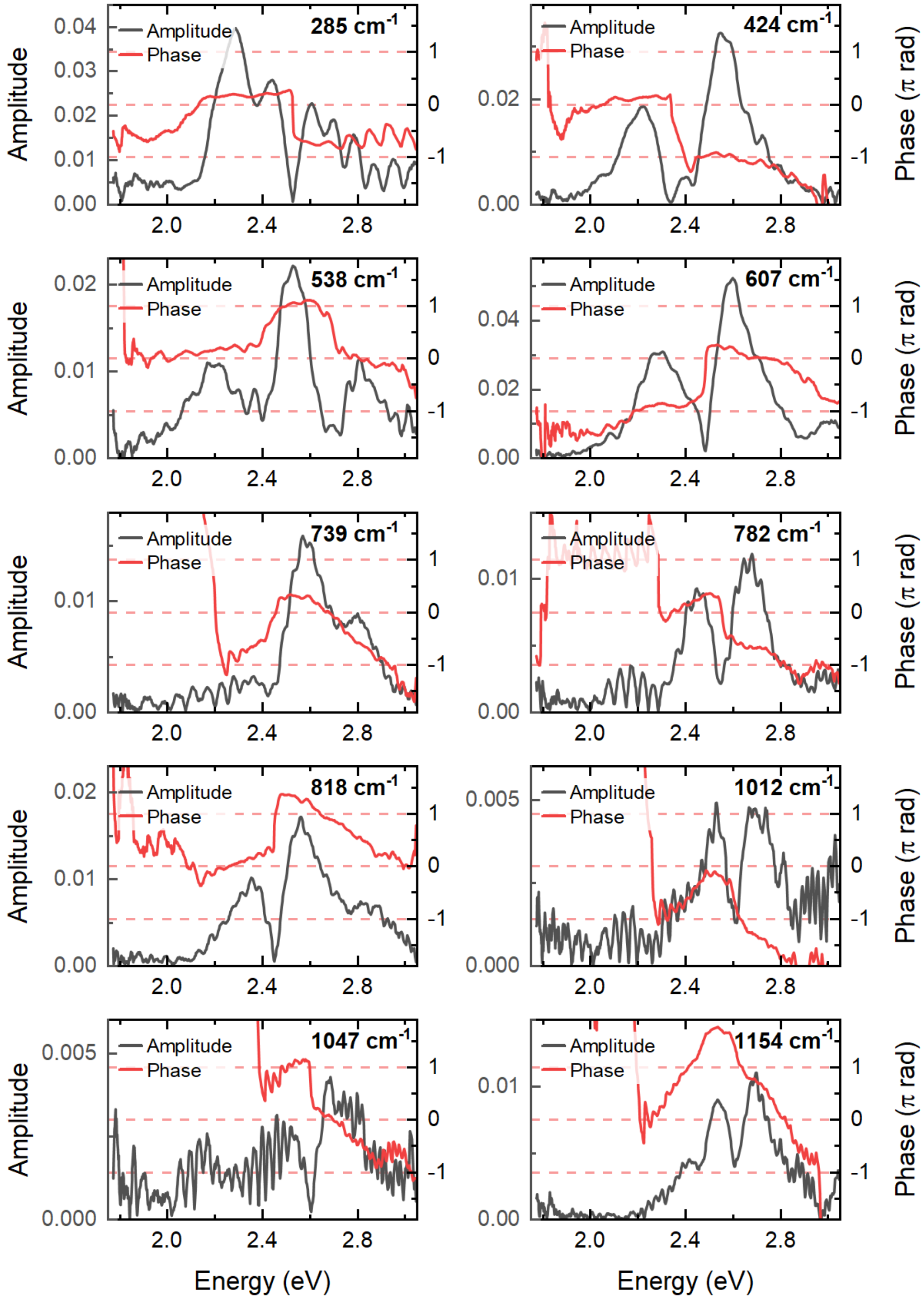

Figure S9: *Spectral mode profiles for selected vibrational modes.*

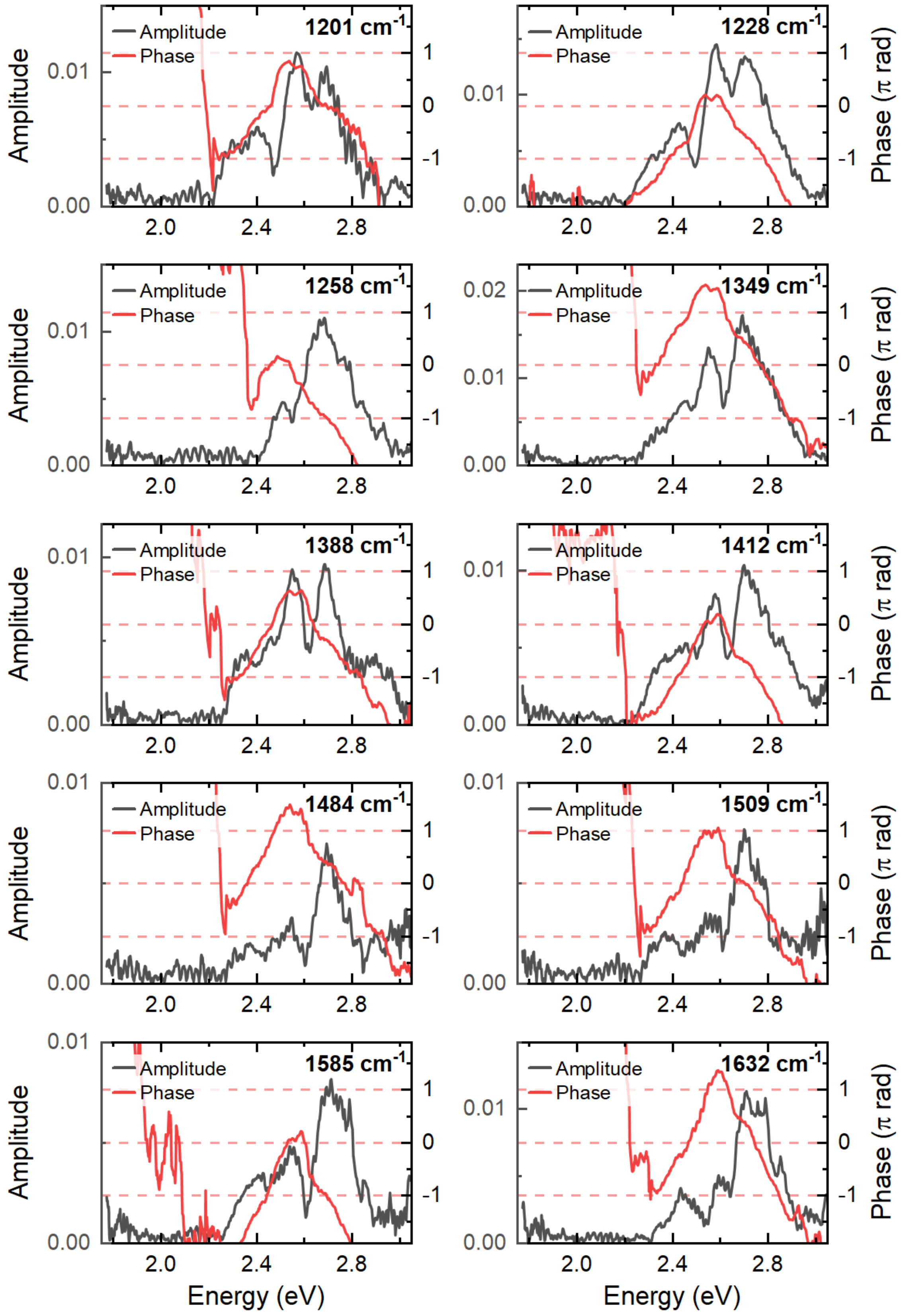

Figure S10: *Spectral mode profiles for selected vibrational modes (continued).*

5. DFT/MRCI calculations

The DFT/MRCI simulation performed on riboflavin to calculate the linear absorption spectrum and excited state spectra at Franck-Condon point (0 fs) and from a relaxed S_1 geometry, for methodological details, see main paper.

Table S2: Linear absorption spectrum calculated for riboflavin using DFT/MRCI; energies scaled by a factor of 0.9.

Linear Absorption		
Transition	dE(eV)	Oscillator Strength
$S_0 \rightarrow S_1$	2.745	0.31616
$S_0 \rightarrow S_2$	3.06	0.0042
$S_0 \rightarrow S_3$	3.366	0.23712
$S_0 \rightarrow S_4$	3.402	0.01309
$S_0 \rightarrow S_5$	3.996	0.00011
$S_0 \rightarrow S_6$	4.077	0.00712
$S_0 \rightarrow S_7$	4.167	0.02948
$S_0 \rightarrow S_8$	4.356	0.00368
$S_0 \rightarrow S_9$	4.473	0.71078
$S_0 \rightarrow S_{10}$	4.653	0.12172
$S_0 \rightarrow S_{11}$	4.779	0.01258
$S_0 \rightarrow S_{12}$	4.986	0.00269
$S_0 \rightarrow S_{13}$	5.121	0.00096
$S_0 \rightarrow S_{14}$	5.175	0.06554
$S_0 \rightarrow S_{15}$	5.211	0.29585
$S_0 \rightarrow S_{16}$	5.31	0.28112
$S_0 \rightarrow S_{17}$	5.382	0.05381
$S_0 \rightarrow S_{18}$	5.499	0.00274
$S_0 \rightarrow S_{19}$	5.535	0.27767
$S_0 \rightarrow S_{20}$	5.724	0.05296
$S_0 \rightarrow S_{21}$	5.76	0.00101
$S_0 \rightarrow S_{22}$	5.805	0.00279
$S_0 \rightarrow S_{23}$	5.886	0.00034
$S_0 \rightarrow S_{24}$	5.922	0.01174

Table S3: The stimulated emission and excited state absorption spectra at the Franck-Condon point and relaxed S_1 state, from DFT/MRCI; energies scaled by a factor of 0.9.

	At FC point		Relaxed S_1	
Transition	dE(eV)	Oscillator Strength	dE(eV)	Oscillator Strength
$S_1 \rightarrow S_0$	-2.745	0.31616	-2.34	0.25477
S_1 --> S_2	0.315	5.00E-05	0.594	0.00034
$S_1 \rightarrow S_3$	0.621	0.00847	0.783	0.01998
$S_1 \rightarrow S_4$	0.657	0.00032	0.873	2.00E-05
$S_1 \rightarrow S_5$	1.251	0.0003	1.431	0.07255
$S_1 \rightarrow S_6$	1.332	0.07278	1.503	0.00095
$S_1 \rightarrow S_7$	1.422	0.06193	1.566	0.04325
$S_1 \rightarrow S_8$	1.611	0.00104	1.809	0.00033
$S_1 \rightarrow S_9$	1.728	0.01678	1.971	0.00368
$S_1 \rightarrow S_{10}$	1.908	0.03772	2.178	0.00108
$S_1 \rightarrow S_{11}$	2.034	0.00228	2.214	0.0316
$S_1 \rightarrow S_{12}$	2.241	0.0008	2.358	0.02636
$S_1 \rightarrow S_{13}$	2.376	0.00014	2.448	0.09965
$S_1 \rightarrow S_{14}$	2.43	0.01283	2.583	0.00549
$S_1 \rightarrow S_{15}$	2.466	0.03963	2.61	0.02031
$S_1 \rightarrow S_{16}$	2.565	0.04532	2.736	0.00873
$S_1 \rightarrow S_{17}$	2.637	0.01763	2.736	0.00027
$S_1 \rightarrow S_{18}$	2.754	0.00035	2.988	5.00E-05
$S_1 \rightarrow S_{19}$	2.79	0.01523	3.051	0.01554
$S_1 \rightarrow S_{20}$	2.979	0.00937	3.123	0.19853
$S_1 \rightarrow S_{21}$	3.015	0.00054	3.177	0.0359
$S_1 \rightarrow S_{22}$	3.06	0.10857	3.186	0.08767
$S_1 \rightarrow S_{23}$	3.141	0.00018	3.231	0.01045
$S_1 \rightarrow S_{24}$	3.177	0.13643	3.321	0.00401

6. Simulation of pump-probe signals

To verify the assignment of modes to predominantly ground or excited state vibrational wavepacket dynamics, we perform simulations of pump-probe signals based on nonperturbatively solving the Lindblad master equation[13, 14] for the time-dependent density matrix $\hat{\rho}(t)$ of our system. For that we employ a phenomenological two-mode displaced harmonic oscillator (2D-DHO) model.[15]

Here, we couple an electronic two-level system with ground state $|S_0\rangle$ and excited state $|S_1\rangle$ with energy separation $\hbar\omega_{el}$ to two modes ($a = 1,2$) with frequencies ω_a. They can be described via a 2D-DHO model

using a Holstein Hamiltonian[15, 16] in the non-displaced basis of harmonic oscillator modes that are centered around displacement zero as

$$H_S = \hbar\omega_{el}|S_0\rangle\langle S_0| + \sum_{a=1,2}\hbar\omega_a b_a^\dagger b_a + \sum_{a=1,2}\frac{\Delta_a}{\sqrt{2}}\hbar\omega_a\left(b_a^\dagger + b_a + \frac{\Delta_a}{\sqrt{2}}\right)|S_1\rangle\langle S_1|. \quad \text{(S2)}$$

Here, $b_a^\dagger$ and b_a represent creation and annihilation operators for mode a, respectively. Linear coupling between the electronic transition and the two independent vibrational modes is introduced by a dimensionless displacement Δ_a along dimensionless nuclear coordinate Q_a. The displacement is related to the Huang-Rhys factor via $S = \Delta^2/2$.

To reduce computational cost, the simulations are carried out in the displaced (eigen-) basis in which the vibrational modes in the S_1 state are centered around $Q_\mathrm{a} = \Delta_\mathrm{a}$. In this basis, the coupling elements between vibronic states in the S_1 state vanish. We label the states as $|e, n_1, n_2\rangle$ with $e = S_0, S_1$ and harmonic oscillator states $|n_a\rangle$ with vibrational quantum numbers $n_a = 0,1,2, \ldots$ In the ground state S_0, the DHO wavefunctions $\psi(Q_1, Q_2)$ are non-displaced and described via the Hermite polynomials.[17] In the excited S_1 state, the wavefunctions $\psi(Q_1 - \Delta_1, Q_2 - \Delta_2)$ are displaced along Q_1 and Q_2 by the respective displacement.

In the displaced basis, the transition dipole moments μ from the ground state S_0 to the excited state manifold now contain the information about the excited state displacement. We compute the transition dipole moment operator $\hat{\mu}$ numerically by using the overlap integrals of the DHO wavefunctions.[17] In this case, the transition dipole moment operator can be obtained from

$$\langle S_0, n_1, n_2|\hat{\mu}|S_1, n_1', n_2'\rangle = \mu_{el}\left\langle\psi(Q_1, Q_2)_{n_1,n_2}\middle|\psi(Q_1 - \Delta_1, Q_2 - \Delta_2)_{n_1',n_2'}\right\rangle \quad \text{(S3)}$$

with electronic transition dipole moment μ_{el}. This shows that the transition dipole moment between two states is given by the overlap integral between the ground and excited state DHO wavefunctions, which square yields the Franck-Condon factors (FCF). We initially prepare the system in the ground state $|S_0, 0,0\rangle$, neglecting any temperature-induced line-broadening effects in the spectra.

Numerical modelling

In a nonperturbative approach,[18-20] we numerically solve the Liouville-von Neumann equation in Lindblad form[13, 14]

$$\dot{\hat{\rho}} = -\frac{i}{\hbar}\left[\hat{H}, \hat{\rho}\right] + \frac{1}{2}\sum_k\left(2\hat{L}_k\hat{\rho}\hat{L}_k^\dagger - \hat{L}_k^\dagger\hat{L}_k\hat{\rho} - \hat{\rho}\hat{L}_k^\dagger\hat{L}_k\right). \quad \text{(S4)}$$

to obtain the evolution of the density matrix $\hat{\rho}$ under the total Hamiltonian $\hat{H} = \hat{H}_S + \hat{H}_{int}(t)$ that is given as the sum of the system Hamiltonian $\hat{H}_S$ and the (time-dependent) light-matter interaction Hamiltonian $\hat{H}_{int}(t)$. System-bath interactions, i.e. vibrational relaxation and electronic dephasing, are accounted for via Lindblad operators $\hat{L}_k$.[13, 14]

Interactions with the two laser electric fields (pump pu and probe pr) are accounted for in dipolar approximation[21, 22] via $\hat{H}_{int} = -\hat{\mu}E(t)$ with total electric field

$$E(t) = \sum_{n=pu,pr} E_{0,n} e^{-2ln2\left(\frac{t-t_n'}{\Delta t_n}\right)^2}\cos(\omega_n(t - t_n') + \phi_n). \quad \text{(S5)}$$

comprising the Gaussian pump and probe with amplitudes $E_{0,n}$, pulse durations Δt_n, phases ϕ_n and carrier frequencies ω_n. The relative delay between pump and probe $t_d = t_{pr}' - t_{pu}'$ marks the pump-probe delay,

while t is the detection time used for numerical integration of Eq. (S4) via 4th order Runge-Kutta in step sizes of 0.1 fs.
From the time-dependent density matrix we then compute the microscopic polarization[21, 22]

$$P(t) = Tr\big(\hat{\mu}\hat{\rho}(t)\big). \tag{S6}$$

from the trace of the expectation value of $\hat{\mu}$. To isolate the nonlinear from the linear ($P^{(1)}(T,t)$) signal contributions contained in the total polarization $P^{tot}(T,t)$, the polarization is calculated for both cases, with and without pump pulse. Via Fourier transform ($\mathcal{F}$) along t, the linear ($\chi^{(1)}$) and total susceptibilities $\chi^{(tot)}$ are obtained: [23]

$$\chi^{(1)}(E_{det}) = \frac{1}{\varepsilon_0}\mathcal{F}\big(P^{(1)}(t)\big)/\mathcal{F}\big(E_{pr}(t)\big) \tag{S7}$$

$$\chi^{tot}(T,E_{det}) = \frac{1}{\varepsilon_0}\mathcal{F}(P^{tot}(T,t))/\mathcal{F}\big(E_{pr}(t)\big). \tag{S8}$$

Here, the signal is normalized to the probe laser spectrum. ε_0 denotes the vacuum dielectric constant.

To account for the directional phase-matching constraints set by the experiment, we employ a 4-step phase-cycling scheme to isolate the desired and relevant third-order contribution from the total nonlinear response using $\phi_{pu} = \left[0,\frac{\pi}{2},\pi,\frac{3\pi}{2}\right]$. After averaging the nonlinear susceptibility for these four phase values, the third-order contribution that is also measured in experiment can be isolated via

$$\chi^{(3)}(T,E_{det}) = \chi^{tot}_{PC}(T,E_{det}) - \chi^{(1)}(E_{det}) \tag{S9}$$

by removing the linear contribution from the total phase-cycled susceptibility $\chi^{tot}_{PC}(T,E_{det})$. The differential spectra $\frac{\Delta T}{T}(T,E_{det})$ in transmission geometry correspond to a measurement of the imaginary contribution of $\chi^{(3)}(T,E_{det})$ and thus the simulated pump-probe signal reads:[13, 14]

$$S_{PP}(T,E_{det}) = \Im\big(\chi^{(3)}(T,E_{det})\big). \tag{S10}$$

Lindblad operators

Electronic dephasing is accounted for using a pure dephasing time of $T_2^* = 1/\gamma_{el}$ where γ_{el} denotes the electronic dephasing rate. The Lindblad operator used is of the form[15]

$$\hat{L}_{dep} = \sqrt{2\gamma_{el}}|S_1\rangle\langle S_1|. \tag{S11}$$

Vibrational relaxation of mode a in the excited state S_1 with rate $\kappa_a = 1/T_{rel,a}$ can be introduced via[15]

$$\hat{L}_{rel} = \sqrt{\kappa_{rel,a}}\hat{b}_a. \tag{S12}$$

In the simulations, vibrational relaxation is either turned off or, when turned on, only accounted for in the electronic excited state and for the high-frequency mode, while no vibrational relaxation is introduced for the low-frequency mode (see below). We also neglect any pure vibrational dephasing and assume zero temperature, i.e. no upwards rates.

Simulation parameters

For the simulations, we choose the parameters listed in Table S2. The electronic transition dipole moment is set to $\mu_{el} = 1$. In the time-domain, a Gaussian 50 fs window is additionally applied. The interaction energy μE that couples the laser electric field to optically allowed transitions is chosen sufficiently weak to only depopulate the ground state by approximately 10^{-4}, ensuring that simulations are performed in the linear regime of third order nonlinearities. The highest value amounts to around 0.7 meV for the $|S_0,0,0\rangle \rightarrow |S_1,2,0\rangle$ transition for the interaction at $t = 0$ with the pump pulse. The probe pulse is chosen to be sufficiently short to provide an adequately broad spectral bandwidth.

Since the simulations are conducted in the displaced basis, even for such large displacement, only 5 vibronic states are used per mode, resulting in a dimensionality of the Hilbert space of 50.

Table S4: Simulation parameters.

Parameter	Value
$\hbar\omega_{el}$	2.6 eV
T_2^*	20 fs
$\hbar\omega_1$	160 meV
$\hbar\omega_2$	74 meV
Δ_1	1.6
Δ_2	1.2
$\hbar\omega_{pump}$	2.64 eV
$\hbar\omega_{probe}$	2.25 eV
Δt_{pump}	12 fs
Δt_{probe}	3 fs
$T_{rel,1}$	20 fs
μ_{el}	1 D

Simulation results

Using the parameters described above, Fig. S11a displays the transition dipole moments between the ground state $|S_0, 0,0\rangle$ and the excited state manifold S_1. Assuming no initial thermal population of higher-lying vibrational states within S_0, we can compute a stick spectrum of the linear absorption proportional to the FCFs using μ^2, as shown in Fig. S11b.

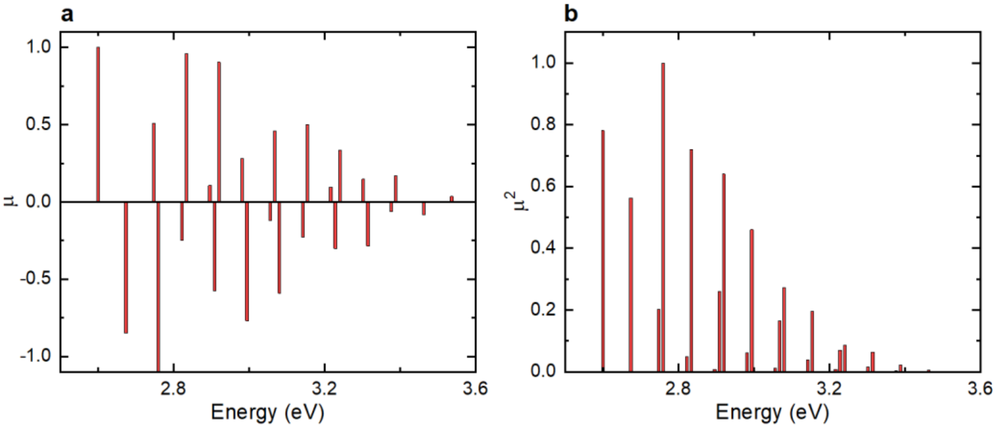

***Figure S11:** Stick spectra for the two-mode DHO. a) Normalized wavefunction overlap between the ground state $|S_0, 0,0\rangle$ and the excited state manifold plotted against the transition energy marking the (normalized) transition dipole moment for optical absorption μ. b) Corresponding FCFs representing the stick spectrum of optical absorption μ^2.*

Even though the experimental data reveal a rich vibrational spectrum containing many low- and high-frequency modes, in this phenomenological model we account for these using an effective 2D-DHO with one low-frequency mode at 600 cm^{-1} and one high-frequency mode at 1300 cm^{-1}. As a result, the displacement chosen for these modes ($\Delta_1 = 1.6, \Delta_2 = 1.2$) is substantially higher than those individual ones we would obtain when considering all experimentally observed modes. In particular, the frequency and displacement of the high-frequency mode are chosen in order to qualitatively reproduce the

experimental absorption spectrum (Fig. S12, red lines). While this simplistic DHO model can qualitatively capture the experimental absorption spectrum, it fails in quantitatively producing the FAD emission spectrum (Fig. S12, cyan lines). Even though there is agreement in overall shape, the experimental emission is substantially more red-shifted by ~200 meV.

Results of the simulation for pump-probe dynamics and spectral mode profiles based on this 2-mode DHO are shown in Fig. S13 for the case of no vibrational relaxation and Fig. S14 with 20-fs vibrational relaxation in the excited state.

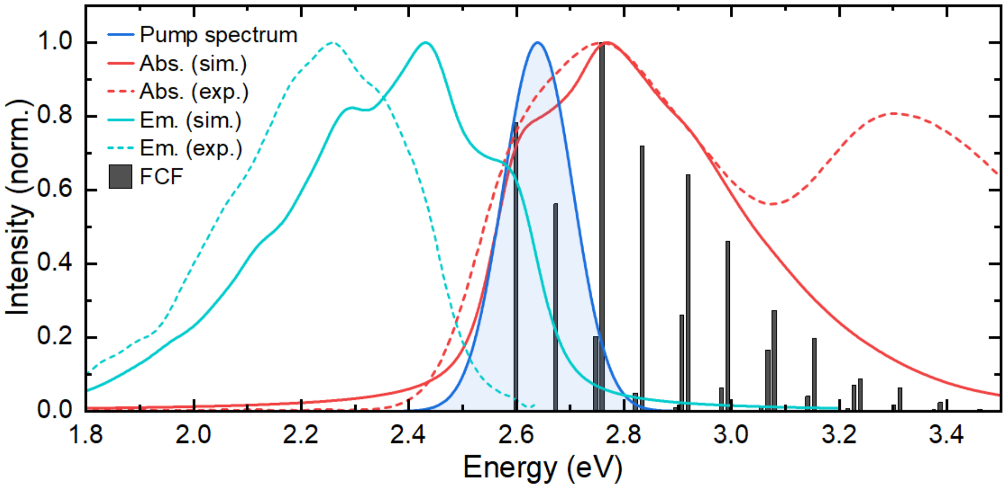

Figure S12: *Simulated absorption spectrum (red solid line) and stick spectrum of the linear absorption from the Franck-Condon factors (black bars) of the 2-mode DHO compared to experimental FAD absorption (red dashed line) and spectrum of the 12-fs pump pulse used in the pump-probe simulations (blue line). The simulated (cyan solid line) and measured (cyan dashed line) emission spectrum is also shown.*

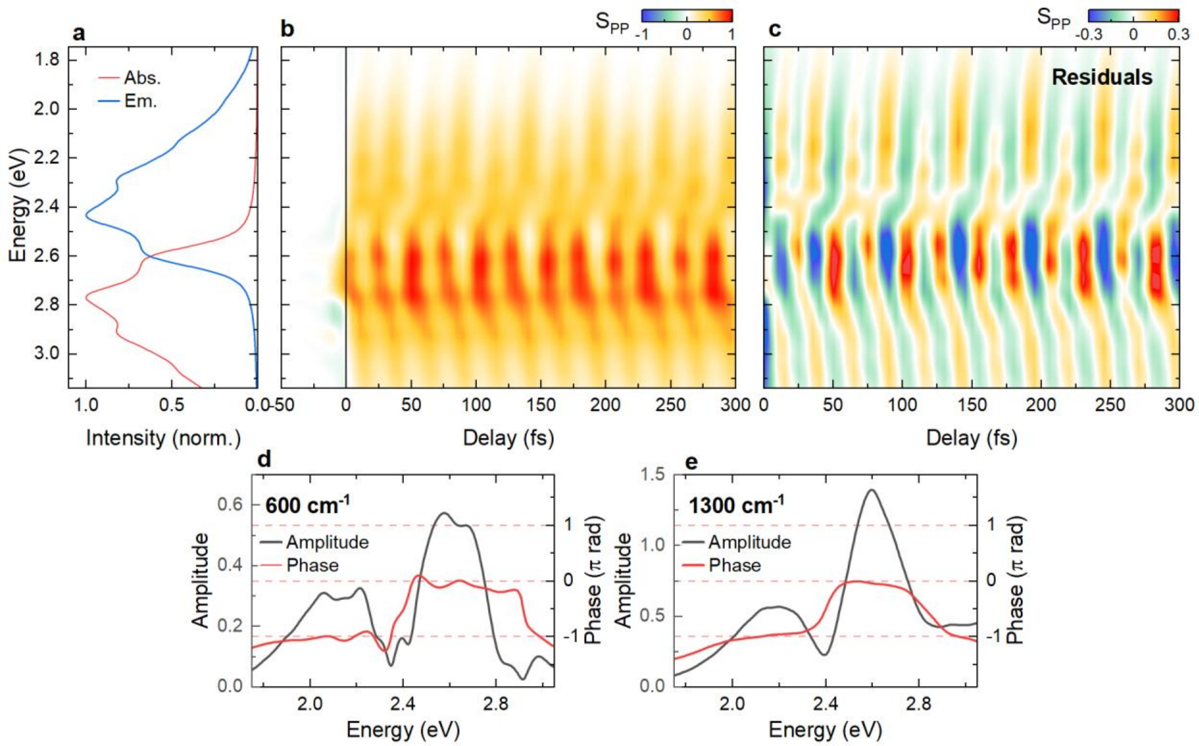

Figure S13: *Simulation results for the 2-mode DHO with a low-frequency 600 cm*$^{-1}$ *and high-frequency 1300 cm*$^{-1}$ *mode without vibrational relaxation. a) Absorption (red line) and emission (blue line) spectra. b) Zoom in of the pump-probe map (computed up to 1 ps). Close to 2.6 eV signal is seen at* $t_d = 0$ *fs, while it takes ~15 fs for the signal to rise in the emission region around 2.2 eV. c) Residuals showing the coherent oscillations. High-frequency oscillations are seen throughout the whole detection energy range. d,e) Spectral mode profiles in amplitude (black lines) and phase (red lines) for the two modes. In both cases, spectral amplitude is seen both in the GSB and SE region with a spectral dip close to 2.4 eV coinciding with a* π *phase jump. The phase is zero at the high energy side of this phase jump.*

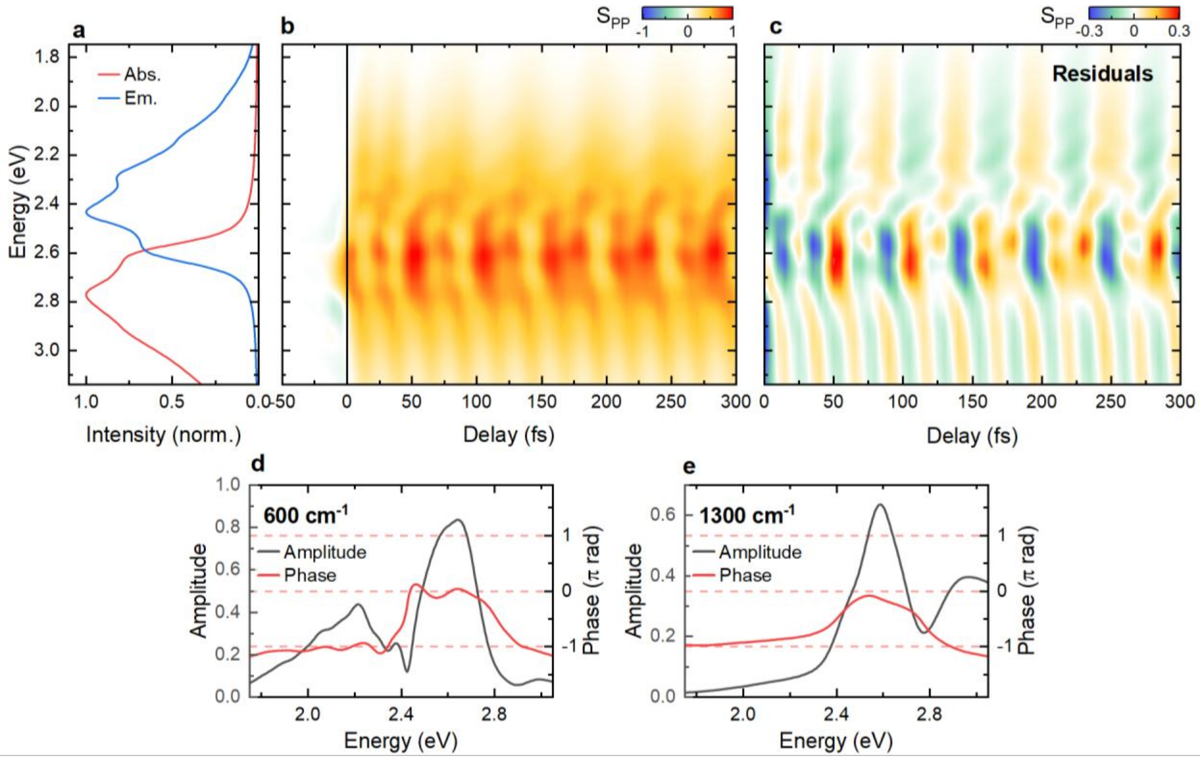

Figure S14: Simulation results for the 2-mode DHO with a low-frequency 600 cm^{-1} and high-frequency 1300 cm^{-1} mode including vibrational relaxation of the high-frequency mode with $T_{rel,1} = 20$ fs. a) Absorption (red line) and emission (blue line) spectra. b) Zoom in of the pump-probe map (computed up to 1 ps). c) Residuals showing the coherent oscillations. High-frequency oscillations are seen now only in the high-energy region, al low energies (~2.2 eV) only the low-frequency mode is seen. d,e) Spectral mode profiles in amplitude (black lines) and phase (red lines) for the two modes. While the low-frequency mode is basically unchanged, the high-frequency mode is now much reduced in amplitude, shows negligible oscillation amplitude in the low-energy region and a smeared out phase profile.

7. References

(1) Timmer, D.; Hergert, G.; Gerhards, L.; Lünemann, D. C.; Schröder, N.; Greven, T.; van der Vlugt, J. I.; De Sio, A.; Solov'yov, I. A.; Christoffers, J. Structural Flexibility Slows Down Charge Transfers in Diaminoterephthalate-C60 Dyads. *The Journal of Physical Chemistry C* **2024**, 2380-2391.
(2) Timmer, D.; Frederiksen, A.; Lünemann, D. C.; Thomas, A. R.; Xu, J.; Bartölke, R.; Schmidt, J.; Kubař, T.; De Sio, A.; Solov'yov, I. A.; et al. Tracking the Electron Transfer Cascade in European Robin Cryptochrome 4 Mutants. *Journal of the American Chemical Society* **2023**, 11566-11578.
(3) Timmer, D.; Lünemann, D. C.; Riese, S.; De Sio, A.; Lienau, C. Full visible range two-dimensional electronic spectroscopy with high time resolution. *Optics Express* **2024**, *32* (1), 835-847.
(4) Macheroux, P. UV-visible spectroscopy as a tool to study flavoproteins. *Flavoprotein protocols* **1999**, 1-7.
(5) Sweetser, J. N.; Fittinghoff, D. N.; Trebino, R. Transient-grating frequency-resolved optical gating. *Optics Letters* **1997**, *22* (8), 519-521.
(6) Ekvall, K.; van der Meulen, P.; Dhollande, C.; Berg, L. E.; Pommeret, S.; Naskrecki, R.; Mialocq, J. C. Cross phase modulation artifact in liquid phase transient absorption spectroscopy. *Journal of Applied Physics* **2000**, *87* (5), 2340-2352.
(7) Rabe, M. Spectram: A MATLAB® and GNU Octave Toolbox for Transition Model Guided Deconvolution of Dynamic Spectroscopic Data. *J. Open Res. Softw.* **2020**, *8*, 13.
(8) Brazard, J.; Usman, A.; Lacombat, F.; Ley, C.; Martin, M. M.; Plaza, P. New Insights into the Ultrafast Photophysics of Oxidized and Reduced FAD ins Solution. *J. Phys. Chem. A* **2011**, *115* (15), 3251-3262.

(9) Copeland, R. A.; Spiro, T. G. Ultraviolet resonance Raman spectroscopy of flavin mononucleotide and flavin-adenine dinucleotide. *The Journal of Physical Chemistry* **1986**, *90* (25), 6648-6654.
(10) Schelvis, J. P.; Pun, D.; Goyal, N.; Sokolova, O. Resonance Raman spectra of the neutral and anionic radical semiquinones of flavin adenine dinucleotide in glucose oxidase revisited. *Journal of Raman Spectroscopy: An International Journal for Original Work in all Aspects of Raman Spectroscopy, Including Higher Order Processes, and also Brillouin and Rayleigh Scattering* **2006**, *37* (8), 822-829.
(11) Merk, V.; Speiser, E.; Werncke, W.; Esser, N.; Kneipp, J. pH-Dependent Flavin Adenine Dinucleotide and Nicotinamide Adenine Dinucleotide Ultraviolet Resonance Raman (UVRR) Spectra at Intracellular Concentration. *Applied Spectroscopy* **2021**, *75* (8), 994-1002.
(12) Weigel, A.; Dobryakov, A.; Klaumünzer, B.; Sajadi, M.; Saalfrank, P.; Ernsting, N. P. Femtosecond Stimulated Raman Spectroscopy of Flavin after Optical Excitation. *Journal of Physical Chemistry B* **2011**, *115* (13), 3656-3680.
(13) Palmieri, B.; Abramavicius, D.; Mukamel, S. Lindblad equations for strongly coupled populations and coherences in photosynthetic complexes. *Journal of Chemical Physics* **2009**, *130* (20), 204512.
(14) Breuer, H.-P.; Petruccione, F. *The theory of open quantum systems*; Oxford University Press, 2002.
(15) Timmer, D.; Zheng, F.; Gittinger, M.; Quenzel, T.; Lünemann, D. C.; Winte, K.; Zhang, Y.; Madjet, M. E.; Zablocki, J.; Lützen, A.; et al. Charge Delocalization and Vibronic Couplings in Quadrupolar Squaraine Dyes. *Journal of the American Chemical Society* **2022**, *144* (41), 19150-19162.
(16) Zhong, C.; Bialas, D.; Collison, C. J.; Spano, F. C. Davydov Splitting in Squaraine Dimers. *The Journal of Physical Chemistry C* **2019**, *123* (30), 18734-18745.
(17) Cohen-Tannoudji, C.; Diu, B.; Laloe, F. Quantum Mechanics, Volume 1. *Quantum Mechanics* **1986**, *1*, 898.
(18) Egorova, D.; Gelin, M. F.; Domcke, W. Analysis of cross peaks in two-dimensional electronic photon-echo spectroscopy for simple models with vibrations and dissipation. *Journal of Chemical Physics* **2007**, *126* (7), 074314.
(19) Seidner, L.; Stock, G.; Domcke, W. Nonperturbative Approach to Femtosecond Spectroscopy - General-Theory and Application to Multidimensional Nonadiabatic Photoisomerization Processes. *Journal of Chemical Physics* **1995**, *103* (10), 3998-4011.
(20) Yan, S. X.; Tan, H. S. Phase cycling schemes for two-dimensional optical spectroscopy with a pump-probe beam geometry. *Chemical Physics* **2009**, *360* (1-3), 110-115.
(21) Hamm, P.; Zanni, M. T. *Concepts and methods of 2d infrared spectroscopy*; Cambridge University Pres, 2011.
(22) Mukamel, S. *Principles of nonlinear optical spectroscopy*; Oxford University Press, 1995.
(23) Boyd, R. *Nonlinear Optics*; Academic Press, 2008 (Third Edition). DOI: https://doi.org/10.1016/B978-0-12-369470-6.00014-9.
